# The Synthesis of Functionalized Carbonized Polymer Dots via Reversible Assembly of Oligomers for Anti‐Counterfeiting, Catalysis, and Gas storage

**DOI:** 10.1002/advs.202405043

**Published:** 2024-08-09

**Authors:** Yu Wang, Yingxi Qin, Fengya Wang, Hongyu Zhang, Changxin Huangfu, Yushu Shi, Xize Chen, Zhenming Wang, Wenming Tian, Liang Feng

**Affiliations:** ^1^ Department of Instrumentation and Analytical Chemistry CAS Key Laboratory of Separation Science for Analytical Chemistry Dalian Institute of Chemical Physics Chinese Academy of Sciences 457 Zhongshan Road Dalian 116023 P. R. China; ^2^ State Key Laboratory of Molecular Reaction Dynamics and the Dynamic Research Center for Energy and Environmental Materials Dalian Institute of Chemical Physics Chinese Academy of Sciences Dalian 116023 P. R. China

**Keywords:** customized functionalization, polymer carbon dots, post‐synthetic doping, selective gas storage, self‐assembly

## Abstract

Carbonized polymer dots (CPDs) have shown exceptional potential across a wide range of applications. However, their practical utilization is significantly greatly impeded by the lack of precise control over their structures and functionalities. Consequently, the development of controlled synthesis strategies for CPDs with well‐defined structures and tailored functionalities remains a critical challenge in the field. Here, the controlled synthesis of functional CPDs with reversible assembly properties via airflow‐assisted melt polymerization, followed by a one‐step post‐synthetic doping strategy, is reported. This synthetic approach achieves high product yield, uniform and tunable structures, as well as customized functionalities including solid‐state emission, enhanced catalytic performance (3.5–45 times higher than conventional methods), and selective gas storage in the resulting CPDs. The ability to tailor the properties of CPDs through controlled synthesis opens up new opportunities for their practical application in photocatalysis and gas storage.

## Graphical abstract

Carbonized polymer dots (CPDs) with well‐defined structures and tailored functionalities are achieved by airflow‐assisted melt polymerization, followed by a one‐step post‐synthetic doping strategy. This synthetic approach achieves high product yield, uniform and tunable structures, as well as customized functionalities including solid–state emission, enhanced catalytic performance, and selective gas storage in the resulting CPDs.

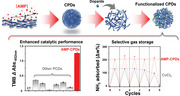

## 1 Introduction

Controlled synthesis of carbon dots (CDs) with well‐defined structures has long been a central focus in the scientific community, as it enables insights into the origin of their diverse functions.^[^ ^1^, ^2^, ^3^, ^4^ ^]^ Among various CDs, Carbonized polymer dots (CPDs) synthesized by botto*m‐*up methods have gained substantial attention due to their exceptional attributes, including high quantum yields,^[^ ^5^, ^6^ ^]^ diverse surface chemistry,^[^ ^2^, ^7^ ^]^ efficient charge separations,^[^ ^8^, ^9^ ^]^ adjustable bandgap,^[^ ^10^, ^11^ ^]^ remarkable chirality.^[^ ^12^, ^13^ ^]^ and minimal toxicity.^[^ ^14^, ^15^ ^]^ These properties make CPDs highly promising for applications in light‐emitting diodes,^[^ ^11^, ^16^, ^17^ ^]^ photocatalysis,^[^ ^18^, ^19^ ^]^ bioimaging,^[^ ^20^, ^21^ ^]^ and clinic therapy.^[^ ^22^, ^23^ ^]^ However, the lack of standardized protocols for CPDs preparation and functionalization often leads to lengthy processes, low yields, uncontrollable properties, and poor reproducibility,^[^ ^3^, ^24^, ^25^ ^]^ thus hindering progress in both fundamental research and practical applications.^[^ ^26^, ^27^ ^]^

The challenges in synthesizing CPDs with well‐defined structures primarily stem from an incomplete understanding of their formation mechanisms.^[^ ^28^ ^]^ Previous studies have suggested that CPDs are formed in a theoretical manner, characterized by a graphitized core with limited extent and a polymeric shell, through the process of initial polymerization followed by concomitant carbonization.^[^ ^2^, ^7^, ^29^ ^]^ However, some CPDs synthesized under mild conditions, where the graphitized core is likely absent or limited due to insufficient carbonization,^[^ ^29^, ^30^ ^]^ also display unique properties that vary with synthesis parameters such as solvent,^[^ ^31^, ^32^, ^33^, ^34^ ^]^ pH,^[^ ^10^, ^35^ ^]^ temperature,^[^ ^36^, ^37^ ^]^ reaction time,^[^ ^38^, ^39^ ^]^ purification,^[^ ^40^, ^41^ ^]^ and precursor activity.^[^ ^3^, ^42^, ^43^ ^]^ This indicates that the unique properties of CPDs arise from a complex mechanism involving various organic reactions that shape their polymer structures and endow them with specific functionalities. For example, our recent work showed that ethanol as a solvent can perplex the structure of CPDs through reactions like esterification, condensation, and catalytic self‐dimerization of precursors.^[^ ^44^ ^]^ This finding underscores the universal existence of unpredicted derivatives within the polymer architectures of CPDs, which synergistically functionalize them with specific properties. Therefore, developing new synthetic methods that effectively mitigate derivative formation is crucial for understanding the origin of specific functionalities and realizing intentional design and customized functionalization in CPDs.^[^ ^45^ ^]^

Here, we report the controlled synthesis of functional CPDs (denoted as TM) through airflow‐assisted melt polymerization (AMP) of tricarballylic acid (TA) and *m‐*phenylenediamine (*m‐*PD), followed by a one‐step post‐synthetic doping strategy. A comprehensive set of characterization techniques, including transmission electron microscopy(TEM), ultra‐performance liquid chromatography quadrupole time‐of‐flight mass spectrometry (UPLC‐QTOF‐MS), nuclear magnetic resonance(NMR), Attenuated total reflection Fourier transform infrared (ATR‐FT‐IR) spectroscopy demonstrate that TM possess relatively homogeneous and adjustable structures compared to CPDs obtained from conventional synthetic methods involving hydrothermal (TM‐H_2_O), solvothermal (TM‐EtOH), and autoclave‐based solvent‐free (TM‐HP) synthesis. Moreover, TM exhibit reversible self‐assembling properties, allowing for the facile incorporation of various dopants, including fluorescent dyes and metal ions, into their polymeric matrix via a one‐step post‐synthetic doping process, thus achieving functionalities such as tunable solid‐state emission (SSE), catalytic performance, and selective gas storage. Our findings not only shed new light on the standardized synthesis of customized functional CPDs on a large scale but also provide a universal platform for investigating diverse interactions, such as charge separation at the interface between CPDs polymer frameworks and different dopants, thereby inspiring the discovery of novel properties in CPDs.

## 2 Results and Discussion

### 2.1 Structural Disparities of CPDs Synthesized by Different Methods

To elucidate the formation mechanism of CPDs, we systematically investigated the synthesis of CPDs by heating TA and *m‐*PD in a 1:1 ratio, with or without the presence of a solvent. Despite the structural similarity between citric acid (CA) and TA, TA was selected as the precursor for CPDs synthesis to circumvent the formation of fluorophore derivatives, such as IPCA (1,2,3,5‐tetrahydro‐5‐oxo‐imidazo[1,2‐α]pyridine‐7‐carboxylic acid), which is commonly observed when using CA.^[^ ^46^, ^47^ ^]^ The lack of a hydroxyl group in TA streamlines its derivatization during the dehydration process, in contrast to CA.^[^ ^30^ ^]^ To mitigate excessive carbonization, the duration of conventional CPDs synthesis methods was restricted to 4 hours, whereas AMP was performed for 2 hours. CPDs synthesized via ethanol‐based solvothermal (TM‐EtOH) and hydrothermal (TM‐H_2_O) methods were purified using conventional dialysis against distilled water. Conversely, CPDs obtained through enclosed solvent‐free autoclave synthesis (TM‐HP) and AMP method (TM) were first dispersed in DMSO solution and subsequently precipitated with methanol, due to their limited dispersibility in most common solvents.

**Scheme 1:**
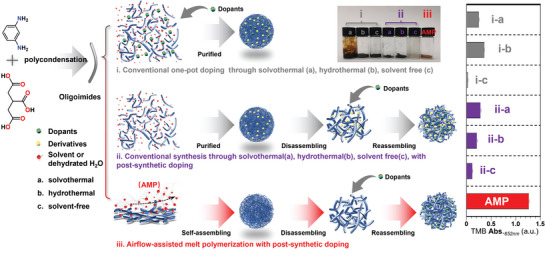
The schematic illustration depicts the distinct synthetic pathways for functionalized CPDs and their corresponding catalytic performance in TMB oxidation (right chart).

**Figure 1:**
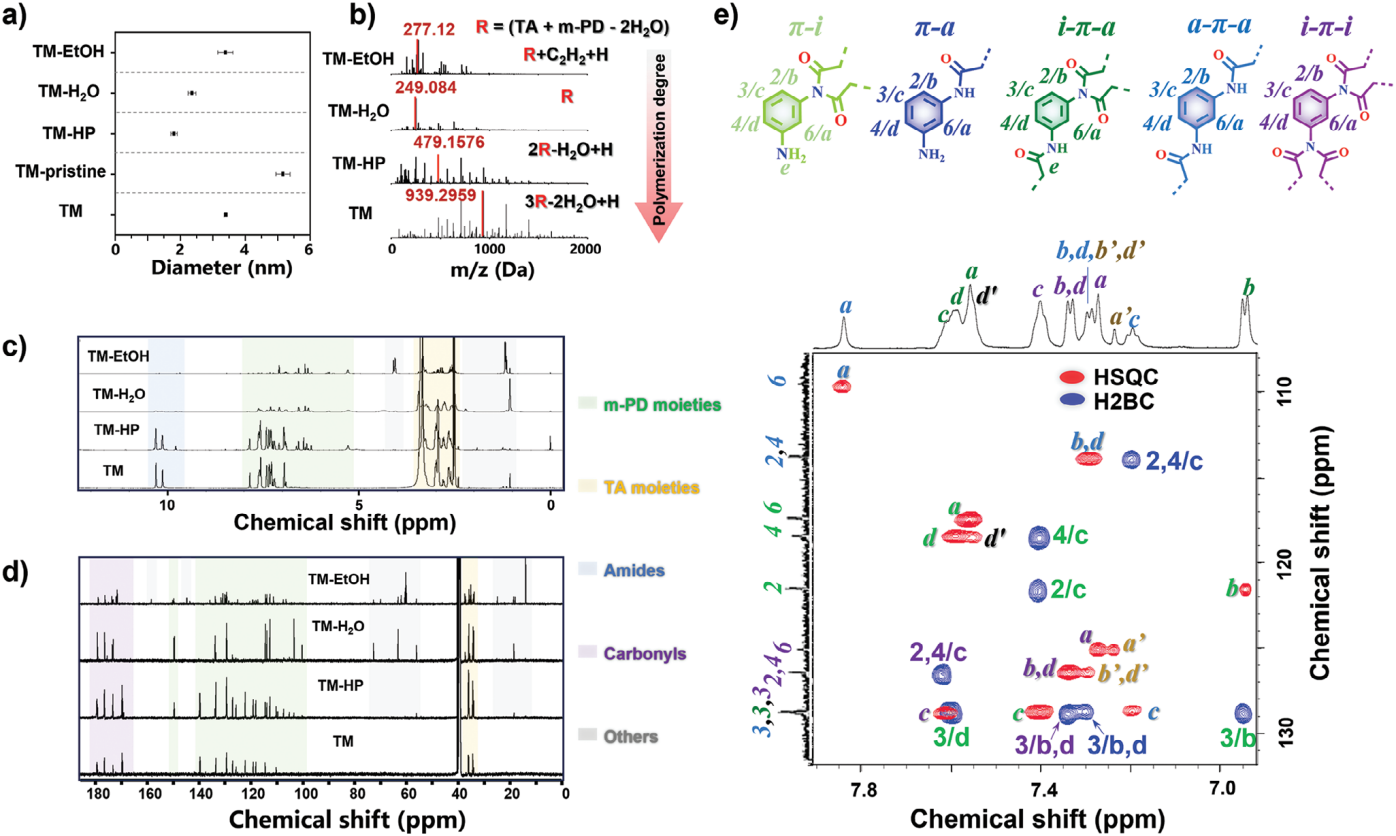
a) The average particle size comparison of different CPDs. b) UPLC‐QTOF‐MS result reveals the identical polymer backbone of four CPDs based on the same molecular formula of R (C_12_H_12_N_2_O_4_). The Comparisons of the c) ^1^H NMR and d) ^13^C NMR spectra show that the main structures of the four CPDs are similar, while TM demonstrates a relatively higher degree of structural homogeneity. e) The phenyl π structure in TM‐pristine. The collaboration between HSQC (red peaks) and H2BC (blue peaks) NMR spectra in phenyl π region reveal distinct C‐H couplings in TM, enabling the identification of five phenyl π structures derived from *m‐*PD: a‐π‐a (blue), a‐π‐i (green), i‐π‐i (purple), a‐π‐i' (black), and i‐π‐i' (dark yellow).

TEM was employed to characterize the morphology of the four synthesized CPDs (**Scheme** **1**; Figure S1a–l, Supporting Information). When dispersed in dimethyl sulfoxide (DMSO) at a concentration of 5 mg mL^−1^, all CPDs displayed a spherical shape with similar particle sizes. Remarkably, the CPDs prepared via AMP (TM) exhibited a relatively monodisperse particle size distribution (**Figure** **1**a; Figure S1j,k, Supporting Information). Interestingly, the average particle size of the raw TM (TM‐pristine) substantially decreased from 5.15 ± 0.22 to 3.40 ± 0.05 nm (TM upon purification, while maintaining its morphology (Figure 1a; Figure S1m–o, Supporting Information). To probe this phenomenon, reverse doping of the precursors into TM (TM‐mix) was performed by co‐dispersing TA and *m‐*PD in DMSO solution and subsequently precipitating with methanol (see Supporting Information for details). The particle size of TM‐mix increased to 4.31 nm (Figure S2, Supporting Information), indicating that unreacted precursors may contribute to the particle size increase, potentially through their assembly onto the TM polymeric frameworks via supramolecular interactions.

UPLC‐QTOF‐MS was utilized to investigate the chemical structure of the CPDs obtained from the different methods. All four products displayed a characteristic molecular formula of C_12_H_12_N_2_O_4_, corresponding to R = (TA + *m‐*PD‐2H_2_O) (Figure 1b). Notably, ion peaks corresponding to *m‐*PD derivatives (CxHyNz species) were observed in all products, with the exception of TM (Tables S1–S3, Supporting Information). Moreover, TM‐EtOH displayed distinct esterification features, corroborating our earlier findings that ethanol is universally involved in the formation of CPDs. Regardless of particle size, the main peaks of TM‐EtOH, TM‐H_2_O, and TM‐HP were found at lower molecular weights region compared to those of TM. Gel permeation chromatography (GPC) experiments further validated this observation, revealing that TM comprises a higher proportion of polymer chains with increased polymerization, as demonstrated by their larger average molecular weights (Mp and Mn) (Figure S3, Supporting Information). These findings suggest that the AMP method promotes polymerization while inhibiting side reactions during synthesis, resulting in the formation of CPDs with comparatively uniform structures.

The NMR analysis was employed to further elucidate the chemical structures of the four CPDs. As illustrated in Figure 1c,d, the ^1^H and ^13^C NMR spectra of TM exhibit similarities to those of TM‐EtOH, TM‐H_2_O, and TM‐HP, albeit with a reduced number of peaks. Heteronuclear single quantum coherence (HSQC) spectra analysis confirmed the presence of *m‐*PD‐derived segments in all CPDs, although in varying proportions (Figure S4, Supporting Information). Interestingly, the proton signals corresponding to terminated *m‐*PD moieties and primary amine (δ from 5.2 to 6.8 ppm) in TM‐EtOH and TM‐H_2_O are more prominent than those associated with intrachain *m‐*PD moieties (δ from 6.9 to 7.9 ppm), indicating the presence of shorter polymer chains in these two CPDs. Moreover, the ^1^H NMR spectrum of TM‐EtOH revealed ethyl ester protons at δ = 1.184 ppm (m) and δ = 4.078 ppm (m, d) with an H integral ratio of 1.53, confirming the occurrence of esterification reactions during synthesis. The combined spectrum of TM‐HP displays features resembling both TM‐H_2_O and TM, suggesting that dehydrated H_2_O can act as a solvent during the synthesis process. For TM, the π protons predominantly correspond to intrachain *m‐*PD‐derived moieties (δ from 6.9 to 7.9 ppm) in the ^1^H NMR spectrum, and the carboxylic C signal is observed at δ = 172.44 ppm in the ^13^C NMR spectrum, indicating that the polymer chains in TM are terminated by carboxylic groups.

Diffusion‐ordered spectroscopy (DOSY) NMR was performed to estimate the particle dimensions of the four CPDs based on their diffusion coefficients using the Stokes‐Einstein relation (Figure S5, Supporting Information).^[^ ^48^, ^49^ ^]^ The DOSY results revealed that TM exhibited a single‐species behavior, whereas the CPDs obtained from conventional methods (i.e., TM‐HP, TM‐H_2_O, and TM‐EtOH) displayed multi‐species characteristics during diffusion. Furthermore, TM was estimated to have larger particle sizes and molecular weights compared to TM‐HP, TM‐H_2_O, and TM‐EtOH. These findings are in agreement with the mass spectrometry results and imply that the polymerization degree of CPDs in solvent‐involved synthesis (e.g., dehydrated H_2_O molecules in TM‐HP) is influenced not only by the condensation reaction equilibrium but also by competing reactions such as esterification, which substantially consumes functional groups, resulting in shorter polymer chains and lower molecular weights in TM‐EtOH, TM‐H_2_O, and TM‐HP. Conversely, AMP method efficiently removes small molecules generated during synthesis, promoting chain growth and yielding relatively uniform structures with a higher degree of polymerization in the polymer chains of TM. Consequently, the relative particle dimensions of TM‐EtOH, TM‐H_2_O, and TM‐HP can be ascribed to the assembly of byproducts formed during synthesis, such as precursors, oligomers, and other species. These results unequivocally establish AMP as an effective method for synthesizing CPDs with high‐quality polymer frameworks.

### 2.2 Characterization and Formation Mechanism of TM Synthesized by AMP Method

To obtain deeper structural insights, 2D NMR analyses, including HSQC, heteronuclear multiple bond correlation (HMBC), and heteronuclear 2‐bond correlation (H2BC), were first performed on both TM‐pristine and TM (Figures S6–S15, Supporting Information). Five types of phenyl amide/imide segments were identified in the pristine TM: aniline‐amide (π‐a), aniline‐imide (π‐i), amide‐phenyl‐amide (a‐π‐a), amide‐phenyl‐imide (a‐π‐i), and imide‐phenyl‐imide (i‐π‐i) (Figure 1e; Figures S6–S10, Supporting Information). Interestingly, the π‐i and π‐a segments were nearly completely absent after purification (Figures S11–S15, Supporting Information), indicating the presence of short‐chain π‐a and π‐i segments. Moreover, HMBC and H2BC NMR analyses of both TM‐pristine and TM revealed that the amide carbonyl carbon is coupled with the methylene protons at δ (2.946, 169.7) ppm and δ (2.924, 168.765) ppm, respectively (Figures S7,S9,S12 and S14, Supporting Information). Given the molecular formula of the dimer unit, R = (TA + *m‐PD –* 2H_2_O), which produces two moles of dehydrated H_2_O, it can be deduced that additional condensation occurs randomly between the carboxylic group at C6 and the amide group at either C1 or C5 (Figure S12, Supporting Information, inset), resulting in the formation of a cyclized succinimide structure with disordered arrangements in the polymer chains. Consequently, the structure of TM was identified as poly (2‐(1‐(3‐aminophenyl)−2,5‐dioxopyrrolidin‐3‐yl) acetic acid), displaying a characteristic over‐dehydration feature. In addition, the HSQC NMR spectrum of TM showed the presence of satellite peaks corresponding to the i‐π‐i' (dark yellow a', b', d') and a‐π‐i' (dark d') moieties (Figure 1e), suggesting that not all imides exist exclusively as succinimides, and differentiated phenyl imides (π‐i') are concurrently formed within TM.

To shed light on the formation mechanism of TM, we synthesized four additional CPDs by varying the molar ratio of TA to *m‐*PD under identical synthetic conditions: TM0.2 (TA: *m‐*PD = 1:5), TM0.4 (TA: *m‐*PD = 1:2.5), TM2.5 (TA: *m‐*PD = 2.5:1), and TM5 (TA: *m‐*PD = 5:1). Compared to TM, TM2.5 and TM5 displayed significant dispersibility in water, requiring a different purification approach that involved dispersing the raw product in acetonitrile followed by precipitation with dichloromethane. The DOSY NMR of as‐obtained TM2.5 also exhibited a single‐species diffusing behavior, indicating the efficacy of the purification process (Figure S16, Supporting Information).

**Figure 2:**
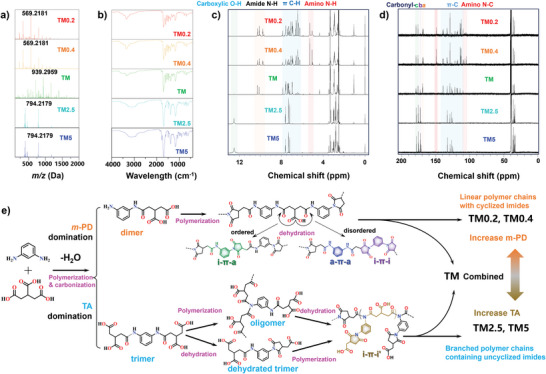
a) UPLC‐QTOF‐MS, b) ATR‐FT‐IR, c) ^1^H NMR and d) ^13^C NMR spectra comparison of TM synthesized using different precursor ratio (*m‐*PD: TA). The structural features of TM0.2 and TM0.4 differ from those of TM2.5 and TM5, while TM exhibits a combination of both sets of structural features. The carbonyl‐a/b/c refer to carbonyl‐a, amide; carbonyl‐b, carboxylic; carbonyl‐c imide. e) Schematic diagram of proposed formation mechanism of TM based on melt‐polymerization. When *m‐*PD is dominant, it results in the growth of polymer chains based on dimers, leading to the production of linear polymers with cyclized imides. Conversely, the dominance of TA leads to the growth of polymer chains based on trimers and yields branched polymers containing uncyclized imide units.

The structural variations among these CPDs were characterized using MS, ATR‐IR, and NMR analyses. MS results showed that TM0.2 and TM0.4 exhibited a characteristic molecular formula of C_30_H_29_N_6_O_6_^+^ (569.2181 Da, *m‐*PD + [(TA + *m‐*PD‐2H_2_O)n – (n‐1)H_2_O]+H)), with a repeating dimer unit (TA + *m‐*PD – 2H_2_O), akin to TM. Conversely, TM2.5 and TM5 displayed a structural feature of C_36_H_36_N_5_O_16_^+^ (794.2179 Da, [(2TA + *m‐*PD – 3H_2_O)n – (n‐1)H_2_O]+H)), suggesting the predominance of a trimer as the repeating unit (2TA + *m‐*PD – 3H_2_O) (**Figure** **2**a). The ATR‐IR spectra of TM0.2 and TM0.4 closely resembled that of TM but differed significantly from those of TM2.5 and TM5 (Figure 2b). Notably, the absence of the amide II vibration peak at 1544 cm^−^1 in TM2.5, along with intensified peaks corresponding to asymmetric and symmetric carbonyl C═O vibrations of imide and imide ring deformation, indicated a decrease in amide groups and an increase in imide groups within the polymer chains of TM2.5 and TM5. Furthermore, a weak and broad band observed in the long‐wavelength region of all three CPDs suggested the presence of hydrogen bonding interactions. Specifically, the absorption band from 2500 to 3420 cm^−^1 in TM2.5 and TM5 could be ascribed to the hydrogen bonding of carboxylic O─H, while the band from 3250 to 3430 cm^−^1 in TM, TM0.4, and TM0.2 was attributed to the hydrogen bonding of amide N─H in TM and TM0.4.

A comparative analysis of the ^1^H and ^13^C NMR spectra of the five CPDs unveiled distinct structural differences among them (Figure 2c,d). Notably, TM0.2 and TM0.4 are characterized by polymer chains terminated by amino groups, as demonstrated by the absence of carboxylic proton signals in the ^1^H NMR spectra and the presence of phenylamine carbon signals in the ^13^C NMR spectra. Conversely, TM2.5 and TM5 are abundant in carboxylic groups but devoid of amino groups in their structures. Moreover, signals corresponding to the amide group are absent in both TM2.5 and TM5, whereas imide‐related carbonyl groups are consistently observed in all CPDs. HSQC, H2BC, and HMBC NMR analyses of TM0.4, TM, and TM2.5 revealed that the splitting C─H coupling signals of methylene within the succinimide ring are consistently observed in all three CPDs (Figures S11–S15 and S17–S22, Supporting Information), indicating the prevalence of succinimide in their structures. Nonetheless, substantial differences are observed in the methylene protons outside the succinimide ring (Figure S23a, Supporting Information). In TM0.4, these protons are observed at δ = 2.97 ppm and exhibit coupling with the amide carbonyl at δ = 168.87 ppm in the H2BC spectrum, akin to TM (Figure S18, Supporting Information). In the case of TM2.5, the methylene protons of cyclized TA shift to δ = 2.79 ppm and exhibit coupling with carboxylic groups at δ = 172.45 ppm, suggesting a distinct C─H coupling (Figure S21, Supporting Information). Furthermore, the proton signals corresponding to phenyl imide in the ^1^H NMR and HSQC spectra of TM0.4 and TM2.5 are identified as i‐π‐i and i‐π‐i', respectively (Figure S23b, Supporting Information). Considering the predominance of amino and amide groups in TM0.4, the characteristic signal of i‐π‐i is likely attributed to meta‐N, N'‐disuccinimidyl benzene, formed by disordered self‐dehydration of TA moieties in the polymer chain (violet structure in Figure 1e).

To gain further insights into the specific structure of i‐π‐i' in TM2.5, an HSQC‐TOCSY (total Correlation Spectroscopy) experiment was performed (Figure S24, Supporting Information). At least three segments derived from TA were identified in TM2.5: an uncyclized TA‐based diimide polymer chain (i), a cyclized TA‐based N‐phenyl‐2‐carboxymethyl‐succinimidyl inner chain segment (ii), and a side group (iii). Remarkably, the absence of asymmetric peaks for the methylene proton belonging to uncyclized TA implies that all terminal TA transformed into N‐phenyl‐2‐carboxymethyl‐succinimidyl groups (iii) in TM2.5. As a result, i‐π‐i' can be assigned to the structure depicted in Figure 2e (khaki color structure). Moreover, the presence of both i‐π‐i and i‐π‐i' in the HSQC spectrum of TM suggests that the initial condensation reaction between *m‐*PD and TA can occur simultaneously in a 1:1 or 1:2 ratio to produce dimer and trimer, unless *m‐*PD is present in a predominant majority.

Based on our findings, the structural formulas of TM0.4 and TM2.5, as well as the formation mechanism of dehydrated‐polymer frameworks, are illustrated in Figure 2e. When *m‐*PD is the dominant precursor, it condenses with TA in a 1:1 ratio to form the TA‐*m‐*PD monoamide dimer, initiating chain propagation. Dehydration occurs through random self‐dehydration at TA moieties, resulting in the formation of three distinct segments: a‐π‐a, a‐π‐i, and i‐π‐i. These polymer chains grow and assemble to form supramolecular polymer frameworks, facilitated by hydrogen bonding interactions. As a consequence, the resulting dehydrated‐polymer frameworks are predominantly terminated by *m‐*PD, resulting in poor dispersibility in most solvents.

In contrast, when TA is present in excess, condensation between TA and *m‐*PD can occur in a 2:1 ratio, yielding a diamide trimer of TA‐*m‐*PD‐TA. Polymer chain propagation proceeds through further condensation between TA carboxylic groups and amide groups from different trimers, leading to the formation of uncyclized imides (π‐i'). At the meantime, unreacted TA in initial trimers undergoes self‐condensation, producing the side group of 2‐carboxymethyl‐succinimide. Chain termination occurs when an i‐π‐i type over‐dehydrated trimer condenses with an amide group. The increased presence of carboxylic groups within the polymer chain significantly enhances dispersibility, allowing the assembled dehydrated‐polymer frameworks of TM2.5 to be dispersed in a wide range of solvents. In summary, the precursor ratio plays a crucial role in governing polymerization pathways during melt polymerization synthesis, ultimately shaping the physicochemical properties of the polymer frameworks in the resulting CPDs.

### 2.3 Self‐Assembling Properties of TM

**Figure 3:**
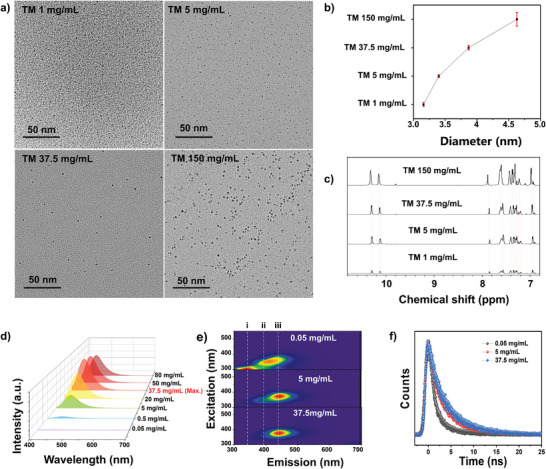
a) TEM images of TM dispersed at different concentrations under the same magnification. b) The diagram of TM average particle size versus dispersing concentration. c) ^1^H NMR spectra of TM at the concentration of 0.05, 1, 37.5, and 150 mg mL^−1^. The concentration‐dependent. d) emission and e) absorption spectrum of TM ranging from 0.01 to 37.5 mg mL^−1^. f) The normalized 3D fluorescence spectra of TM at 0.05, 5, and 37.5 mg mL^−1^, revealing three distinct emission centers (i, ii, and iii). g) TCSPC fluorescence decay of TM at the wavelength of 441 nm measured across various concentrations.

To verify the self‐assembly theory provided in the formation mechanism, rather than aggregation or covalently linkage, characterization of TM at different concentration was implemented. TEM analysis of TM at varying concentrations revealed that particle size increased with the concentration of TM in DMSO solutions used for dispersion on the grid (**Figure** **3**a,b; Figure S25, Supporting Information). Specifically, the average particle diameter of TM increased from 3.16 ± 0.07 nm at a concentration of 1 mg mL^−1^ to 3.87 ± 0.05 nm at a concentration of 37.5 mg mL^−1^, displaying a uniform distribution and excellent dispersion characteristics. Nevertheless, when the concentration was increased to 150 mg mL^−1^, significant particle aggregation was observed, leading to an increase in particle size to 4.36 ± 0.24 nm, accompanied by a broader size distribution (Figure 3b; Figure S25j–l, Supporting Information). This phenomenon was also observed in TEM of TM0.4 and TM2.5 (Figure S26, Supporting Information). To confirm this, dynamic light scattering (DLS) analysis was carried out. An increase in TM particle size from 3.37 to 4.98 nm was determined as the concentration increased in DLS spectrum (Figure S27, Supporting Information). The particle size distribution also broadened significantly with increasing concentration, which is also in agreement with the TEM observations. Furthermore, the ^1^H NMR spectrum revealed that proton signals corresponding to phenyl amide segments exhibited minimal changes in their chemical environment from a concentration of 1–37.5 mg mL^−1^ (Figure 3c), suggesting that the increase in particle size had only a minor impact on polymer chain (i.e., phenyl amides) possibly due to under the same intermolecular force. However, these signals noticeably shifted downfield upon reaching a concentration of 150 mg mL^−1^, indicating the presence of additional interactions that significantly enhance the stability of the polymer chain. Moreover, UPLC‐QTOF‐MS and GPC experiments using a dilute sample at a concentration of 1 mg mL^−1^ demonstrated that the main ion peaks in the MS spectrum were identical, and the average molecular weights (Mw and Mn) were comparable to those at 5 mg mL^−1^ (Figure S28, Supporting Information). Interestingly, the peak average molecular weight (Mp) substantially decreased from 3345 Da at 5 mg mL^−1^ to 2236 Da at 1 mg mL^−1^, indicating an increase in particles with lower molecular weights but minimal changes in their intrinsic structures. These findings unequivocally demonstrate the self‐assembling properties of TM, rather than aggregation. Notably, to further demonstrate the reversibility of self‐assembly, we compared the TEM images and particle size distributions of TM prepared directly at 5 mg mL^−1^ (3.40 nm in Figure S25c, Supporting Information) and TM initially prepared at 150 mg mL^−1^ followed by dilution to 5 mg mL^−1^ (3.45 nm in Figure S29, Supporting Information). No significant differences in particle sizes were observed between the two samples, providing strong evidence for the reversible self‐assembly behavior of TM.

In addition, TM displayed an evident enhanced emission phenomenon as its concentration in the DMSO solution increased (Figure 3d; Figure S30a, Supporting Information). The emission intensity of TM was initially negligible in dilute solutions, but substantially increased with rising concentration, reaching a maximum at 37.5 mg mL^−1^. The absorption spectrum revealed that the initial absorption band of TM, spanning from 250 to 270 nm at a concentration of 0.05 mg mL^−1^, extended into the visible region when the concentration reached 5 mg mL^−1^ (Figure S30b, Supporting Information). This was further confirmed by the concentration‐dependent excitation spectrum of TM (Figure S30c, Supporting Information). The normalized 3D emission spectra for TM at different concentrations depicted the presence of three distinct emission centers (i, ii, iii) at a concentration of 0.05 mg mL^−1^ (Figure 3f). Interestingly, as the concentration of TM increased to 5 and 37.5 mg mL^−1^, the number of emission centers reduced to two and one, further proving that self‐assembly can enhance electronic transitions in the longer wavelength region. Moreover, time‐correlated single‐photon counting (TCSPC) results demonstrated a marked prolongation of the fluorescence lifetime (τ) for TM with increasing concentration in the solution. The τ_441_ measured at 0.05 mg mL^−1^ was 2.85 ns, which significantly prolonged to 4.45 ns at 5 mg mL^−1^ and further extended to 4.85 ns at 37.5 mg mL^−1^ (Figure 3g). As reported in the literature,^[^ ^50^ ^]^ changes in fluorescence lifetime are closely related to the regulation of nonradiative processes. Therefore, it can be inferred that self‐assembly increases the rigidity of the system, effectively suppressing nonradiative relaxation processes, thereby enhancing the fluorescence intensity and lifetime of TM in the visible region.

To elucidate the underlying mechanism of self‐assembly‐enhanced emission, we investigated the changes in TM properties using different binary solutions. Water (H_2_O) and chloroform (CHCl_3_) were added to DMSO solutions in varying proportions to assess their impact on self‐assembly. The addition of H_2_O strengthened interchain hydrogen bonding interactions, whereas the presence of CHCl_3_ exerted a negative effect on self‐assembly.^[^ ^51^ ^]^ Although the addition of both H_2_O and CHCl_3_ diminished the dispersibility of TM in the DMSO solution, the emission of TM was affected differently. With increasing H_2_O content in DMSO, the emission initially intensified but subsequently declined when the proportion of H_2_O reached 40% (Figure S31a, Supporting Information). In contrast, a sustained decrease in emission was observed upon the addition of CHCl_3_. TEM images clearly showed that in the presence of 10% water, larger particles with an average size of 29 nm were observed. In contrast (Figure S31b, Supporting Information), while CHCl_3_ only induced apparent particle aggregation without significant changes in particle size and morphology (Figure S31c, Supporting Information). Furthermore, temperature‐dependent emission experiments revealed that increasing the percentage of H_2_O in the DMSO solution alleviated the decrease in emission intensity upon heating (Figure S32, Supporting Information). This suggests that reinforcing hydrogen bonding interactions effectively improves the structural stability of self‐assembly, thereby considerably mitigating the emission quenching induced by heating. Consequently, the enhancement of hydrogen bonding interactions promotes assembling properties, resulting in increased rigidity of frameworks and enhanced emission. Conversely, the presence of CHCl_3_ hinders the assembling behavior of the polymer chain, leading to particle aggregation and a reduction in emission intensity. The ^1^H NMR and HSQC results revealed a remarkable upfield shift in the protons associated with phenyl amide groups, signifying a substantial perturbation of the chemical environment upon exposure to water (Figure S33, Supporting Information). In contrast, a downfield shift of these protons was observed upon the addition of chloroform, implying the occurrence of particle aggregation similar to that observed at a concentration of 150 mg mL^−1^ in Figure 3c.

To gain insights into the kinetics of self‐assembly and the origin of self‐assembly‐enhanced emission in CPDs, density functional theory (DFT) calculations were performed.^[^ ^30^ ^]^ The self‐assembling behavior was investigated by optimizing the structures of two types of a‐π‐i tetramers (Figure S34a, Supporting Information). The visual representation of the reduced density gradient (RDG) isosurface unequivocally confirmed the presence of supramolecular interactions, such as interchain hydrogen bonding between amide N─H and imide C═O groups, as well as π–π stacking between terminal phenyl rings (Figure S34b, Supporting Information). To investigate the emission phenomena, DFT calculations were performed on three nonbranched π‐segments (a‐π‐i, i‐π‐i, and a‐π‐a) and two branched segments (i‐π‐i' (i) and (ii)) to simplify the analysis (Figure S34c, Supporting Information). The results revealed that exciton relaxation in all segments for emission occurs through the lowest unoccupied molecular orbital (LUMO) and highest occupied molecular orbital (HOMO), which are primarily contributed by the succinimide and phenyl ring. Notably, the oscillator strengths of all structures were found to be below 0.1 (Table S4, Supporting Information), which is consistent with the experimental observation of negligible fluorescence at low concentrations. These DFT calculations corroborate the experimental findings, indicating that self‐assembly, driven by supramolecular interactions such as hydrogen bonding and π–π stacking, substantially enhances the emission of CPDs in the visible region.

### 2.4 Functionalization of TM and Their Applications

**Figure 4:**
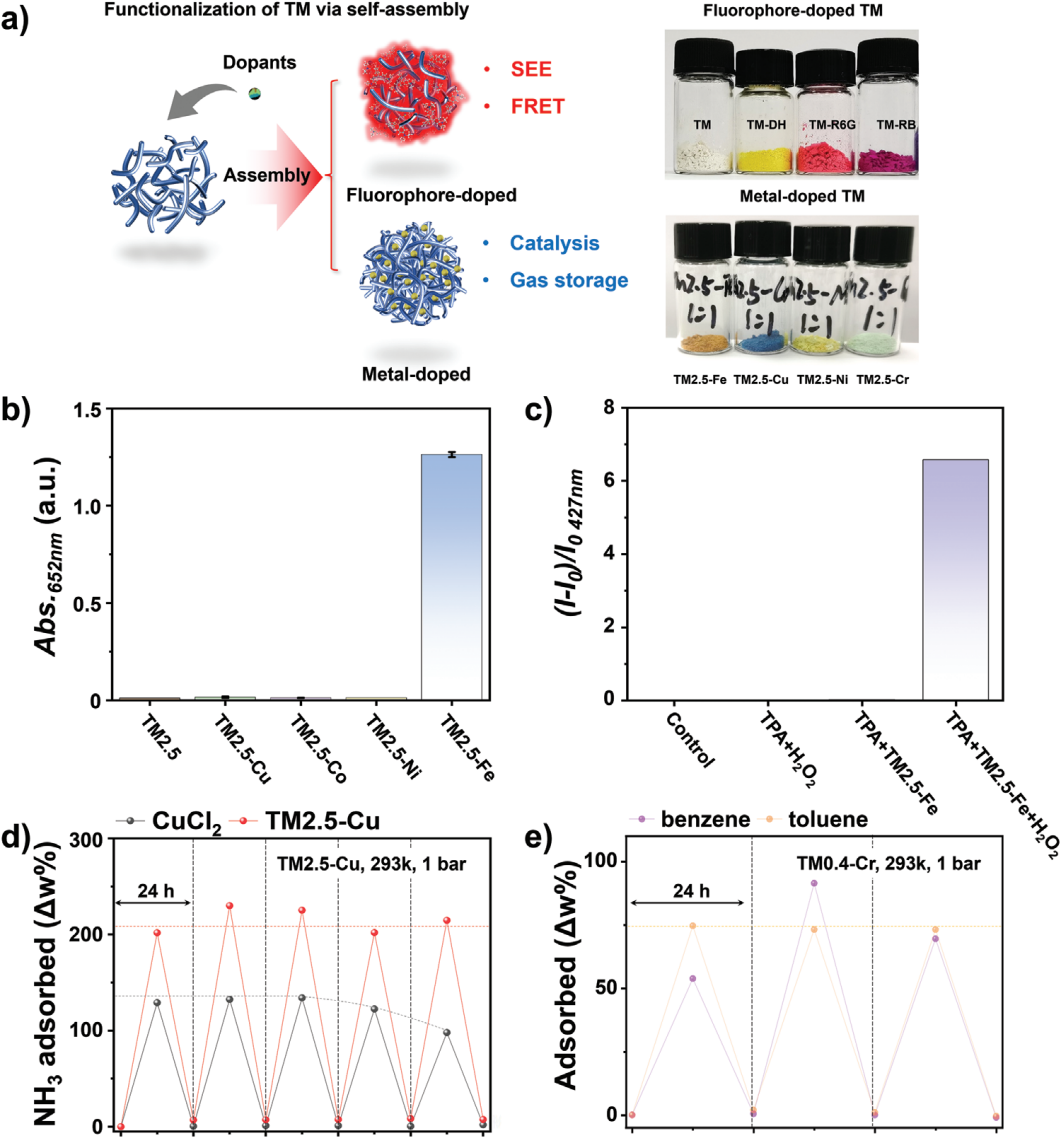
a) The schematic illustration of customized functionalization of CPDs by one‐step post‐synthetic doping using different dopants based on self‐assembly. The right photographs show the product CPDs. b) The catalytic performance of various metal‐doped TM2.5 synthesized using the AMP method was evaluated by measuring the increase in absorbance at 652 nm of a 1 mm TMB aqueous solution. c) The fluorescence intensity at 427 nm of a 5 mmol TPA aqueous solution was measured in the presence of 0.05 mg mL^−1^ TM2.5‐Fe. d) Reversible storage of NH_3_ was achieved utilizing hydrophilic TM2.5‐Cu under ambient conditions without any additional treatment. e) Hydrophobic TM0.4‐Cr demonstrated reversible storage of benzene and toluene under ambient conditions.

The reversible assembling property of TM enabled the facile functionalization of TM through co‐dispersion of different dopants followed by their incorporation (**Figure** **4**a). Various fluorescent molecules, including rhodamine B (RB), rhodamine 6G (R6G), and 3,6‐diaminoacridine hydrochloride (DH), were initially doped using this method. The resulting CPDs exhibited remarkable SSE (Figure S35a,b, Supporting Information), surpassing those obtained from conventional one‐pot doping methods. For example, TM‐RB exhibited strong SSE, while TM‐EtOH‐RB, TM‐H_2_O‐RB, and TM‐HP‐RB displayed weak or no emission (Figure S35c, Supporting Information). The spectral overlap between TM emission and the absorption of fluorescent dyes (Figure S35d, Supporting Information), along with the decreased lifetime of TM at 441 nm after doping (Figure S35e, Supporting Information), provided evidence for intraparticle fluorescence resonance energy transfer (FRET). Interestingly, the as‐fabricated CPDs exhibited distinctive SSE under different excitation wavelengths. When a pentapetalous flower pattern was depicted using 10 mg mL^−1^ DMSO solutions of TM, TM‐RB, TM‐R6G, and TM‐DH (Figure S35f, Supporting Information), it displayed distinct appearances under different excitation wavelengths: a quatrefoil pattern under 254 nm excitation, a cinquefoil pattern under 365 nm excitation, and a red quatrefoil pattern under 410 nm excitation (top right pictures). Furthermore, the inherently poor dispersibility of TM in commonly used solvents such as water, acetonitrile, and toluene, led to the preservation of this pattern even when immersed in these solvents (bottom right pictures). This highlights the potential application of fluorophore‐doped TM as robust fluorescent materials for anti‐counterfeiting purposes.

Additionally, metal‐doped CPDs, including TM2.5‐Co, TM2.5‐Ni, TM2.5‐Cu, and TM2.5‐Fe, were investigated to explore their catalytic oxidation performance. Upon the addition of 0.05 mg mL^−1^ of these metal‐doped TM2.5 into a 1 mm aqueous tetramethylbenzidine (TMB) solution under dark ambient conditions, the absorbance at 652 nm was measured after 10 min. Remarkably, TM2.5‐Fe demonstrated outstanding catalytic properties, inducing the increase of TMB absorbance from 0 to 1.26 without any additional stimulation (Figure 4b). Compared to other Fe‐doped CPDs obtained through conventional methods mentioned in Scheme 1, TM2.5‐Fe exhibited significantly enhanced catalytic capability (Figure S36a, Supporting Information), thereby highlighting the effectiveness of post‐synthetic doping strategies based on reversibly assembling CPDs for functionalization purposes. By further adjusting the doping concentration of Fe^3+^ within a range from 2 mmol g^−1^ (TM2.5‐Fe0.3) to 6 mmol g^−1^ (TM2.5‐Fe), the oxidation rate of the TMB exhibited significant enhancement (Figure S36b, Supporting Information), emphasizing that post‐synthetic doping based on self‐assembly can also tune the catalytic properties of CPDs. Moreover, upon the addition of H_2_O_2_, TM2.5‐Fe were capable of oxidizing terephthalic acid(TPA) without further treatments(Figure 4c; Figure S36c, Supporting Information), as the fluorescence intensity at 427 nm of TPA was dramatically enhanced, indicating their high potential in producing OH^•−^ for self‐catalytic oxidation reactions.

The search for innovative gas storage materials is critical for advancing clean energy technologies, improving the efficiency and safety of gas storage, enabling new applications, and addressing key energy and environmental challenges. Using metal‐doped TM, we demonstrated the outstanding potential of TM‐based CPDs in the application of selective gas storage, which has never been reported in the field of CDs previously. By doping Cu^2+^ into hydrophilic TM2.5, TM2.5‐Cu enables reversible adsorption and release of ammonia at ambient temperature and pressure. As shown in Figure 4d, when using 20 mg of TM2.5‐Cu and 20 mg of CuCl_2_ in a saturated ammonia atmosphere (293 K, 1 bar), TM2.5‐Cu exhibited superior ammonia adsorption capacity, with the adsorbed NH_3_ weight exceeding 200% of its own weight. Furthermore, the performance of TM2.5‐Cu remained stable after five adsorption–release cycles. In contrast, CuCl_2_ showed an adsorption capacity of only ≈130% of its own weight and a significant performance decay after merely three cycles. Additionally, by doping hydrophobic TM0.4 with Cr^3+^, the resulting TM0.4‐Cr achieved reversible adsorption of gaseous toluene and benzene (293 K, 1 bar), with an adsorption capacity reaching 75% of its own weight (Figure 4e). In particular, TM0.4‐Cr exhibited better adsorption stability for benzene, which may be attributed to the higher affinity between Cr^3+^ and benzene, such as in bis(benzene)chromium. These findings demonstrate that TM, as porous supramolecular self‐assembling materials with tunable chemical structures, can achieve highly efficient and stable gas storage through the selective doping of metal ions. The ability to tailor the gas adsorption properties of TM through metal ion doping opens up new possibilities for the development of advanced gas storage materials. Further investigation into the underlying mechanisms and the optimization of the doping strategy may lead to the development of even more efficient and selective gas adsorbents based on the TM platform.

## 3 Conclusion

In summary, we have developed a novel method for scalable and controlled synthesis of functional carbonized polymer dots (CPDs). This method involves airflow‐assisted melt polymerization (AMP), followed by post‐synthetic doping via reversible self‐assembly. The intrinsic CPDs obtained from AMP exhibit relatively uniform structures and excellent self‐assembling properties. A comprehensive suite of characterization techniques, including NMR, MS, TEM, and DFT calculations, provide profound insights into the formation mechanism of CPDs. These investigations reveal that the precursor ratio plays a pivotal role in directing the polymerization pathways and ultimately determining the physicochemical properties of the resulting polymeric frameworks. Notably, leveraging the inherent self‐assembling behavior of CPDs enables the facile incorporation of various dopants, such as fluorescent dyes and metal ions, into their polymeric framework via post‐synthetic doping. This strategy allows for the customization of CPDs' properties, leading to tunable solid‐state emission, excellent self‐catalytic performance, and selective gas storage capabilities. The ability to tailor functionality through self‐assembly‐based post‐synthetic doping opens up new avenues for the development of advanced nanomaterials with unique properties. Our findings not only deepen our understanding of structure–property relationships in CPDs but also highlight their immense potential as versatile nanoplatforms for diverse applications ranging from optoelectronics and catalysis to gas storage.

## 4 Experimental Section

### Chemicals

*m‐*phenylenediamine (*m‐*PD), tricarballylic acid (TA), acetonitrile, DMSO, methanol, ethanol, dichloromethane, and chloroform were purchased from Macklin Reagent (Shanghai) Co. Ltd., 3,6‐Diaminoacridine Hydrochloride was purchased from Adamas (Shanghai). Dimethyl sulfoxide‐*d*6 (DMSO*‐d*6) was purchased from Sigma–Aldrich. All chemicals were used without further purification.

### Synthesis—Synthesis of TM‐EtOH and TM‐H_2_O

To a 20 mL glass vial, 0.2 mmol TA and 0.2 mmol *m‐*PD were added into 10 mL ethanol and sonicating for 10 min. The mixture was then transferred to a 50 mL Teflon‐sealed autoclave. The autoclave was heated to 230 °C and kept for 4 h. After reaction, the autoclave was cooled down to room temperature. The product was transferred to 10 mL dialysis kit (0.5–1 kDa molecular weight cut off, Float‐A‐Lyzer G2, Spectrum) and dialyzed against distilled water in 2 L beaker for 48 h (changing water for every 12 h). The as‐obtained product was then separated by centrifugation at 10 000 rpm (11 952 g) for 10 min. The supernatant was further purified by filtering with 0.22 µm filter membrane (Polyesthersulfone) and then collected as TM‐EtOH. The TM‐H_2_O were synthesized using H_2_O as the solvent in the same procedure.

### Synthesis—Synthesis of TM‐HP

The *m‐*PD and TA were added to a mortar in equimolar ratios, thoroughly mixed, and homogenized before being transferred to a 50 mL Teflon‐sealed autoclave. The autoclave was then heated to 230 °C and maintained at this temperature for 2 h. Following the reaction, the autoclave was cooled down to room temperature. The purification process employed was identical to that of TM.

### Synthesis—Synthesis of TM, TM0.4 and TM0.2

Equimolar ratios of *m‐*PD and TA were added to a mortar, mixed thoroughly, and ground evenly before being transferred to a quartz boat. The quartz boat was then placed in a tube furnace. After 20 min of nitrogen purging, the reaction mixture was heated to 230 °C for 2 h. Subsequently, the raw product was cooled to room temperature and dispersed into DMSO with the assistance of ultrasound (using a product‐to‐solvent weight ratio of 1:20) followed by stirring for 10 min. Methanol was subsequently added for precipitation (EtOH:DMSO = 10:1 v/v). The precipitate was filtered and washed three times with water and Methanol consecutively. Finally, TM were obtained as a white powder immediately after drying. The yield was calculated ≈75% (w/w). The TM0.4 was synthesized in the same course, with a molar ratio of *m‐*PD to TA of 2.5:1. The yield ofTM0.4 was calculated ≈71% (w/w).

### Synthesis—Synthesis of TM2.5 and TM5

The *m‐*PD and TA were combined in a molar ratio of 1:2.5, thoroughly mixed, and finely ground before being transferred to a quartz boat. Subsequently, the quartz boat was placed inside a tube furnace. Following 20 min of nitrogen purging, the reaction mixture was heated to 230 °C for 2 h. Afterward, the resulting raw product was cooled to room temperature and pulverized into powder before being transferred into a 50 mL round flask. Due to the excellent dispersibility of the raw product in most polar solvents, a modification was made to the purification process by employing a binary solvent system with lower polarity, i.e., acetonitrile‐dichloromethane. Particularly, 20 mL of acetonitrile was added and stirred for 10 min. Next, an additional 30 mL of dichloromethane was added. The precipitate was filtered and washed three times with dichloromethane. Finally, TM2.5 were obtained after vacuum drying at 30 °C for 6 h. The yield was calculated ≈73% (w/w).

### Synthesis—Fabrication of TM‐mix

The TM, TA, and *m‐*PD were individually weighed at 0.15 g each and dissolved in 2.7 mL of dimethyl sulfoxide (DMSO). Subsequently, the mixture was sonicated for 5 min and magnetically stirred for 30 min before being supplemented with 30 mL of methanol, resulting in significant precipitation. After filtration with three subsequent washes using methanol and water, respectively. The product was subjected to vacuum drying at a temperature of 60 °C for 6 h to obtain TM‐mix powders.

### Synthesis—Post‐Synthetic Doping of TM with Metal Ions

The TM and transition metal chloride, weighing 0.15 g each, were separately dissolved in 2.7 mL of dimethyl sulfoxide (DMSO) and 0.3 mL of water, respectively. Subsequently, the two solutions were mixed and sonicated for a duration of 5 min. The resulting mixture was magnetically stirred for a period of 30 min before being supplemented with 30 mL of distilled water, leading to the formation of substantial precipitation. After completing filtration with three subsequent washes using water, the product was subjected to vacuum drying at a temperature of 60 °C for 6 h to obtain metal‐doped TM powders including TM‐Fe, TM‐Co, TM‐Cr, TM‐Ni, and TM‐Cu.

### Synthesis—Post‐Synthetic Doping of with Fluorophores

TM (0.15 g) and fluorescent dyes (12 mg) were individually weighed, followed by their dissolution in 2.7 mL of dimethyl sulfoxide (DMSO) and 0.3 mL of methanol, respectively. Subsequently, the two solutions were combined and sonicated for a duration of 5 min. The resulting mixture was magnetically stirred for a period of 30 min before being supplemented with 30 mL of methanol, leading to the formation of significant precipitation. After undergoing filtration with three subsequent washes using methanol, the product was subjected to vacuum drying at a temperature of 60 °C for 6 h to obtain fluorophore‐doped TM powders including TM‐DH, TM‐R6G, TM‐RB, and TM‐NR.

### Synthesis—Synthesis of TM‐EtOH‐Fe, TM‐H_2_O‐Fe and TM‐HP‐Fe

The synthesis methods for TM‐EtOH‐Fe, TM‐H_2_O‐Fe, and TM‐HP‐Fe are identical to those for TM‐EtOH, TM‐H_2_O, and TM‐HP, respectively, with the sole distinction being the inclusion of equimolar quantities of ferric chloride in the precursors. All other procedures remain unaltered.

### Characterization—UPLC‐QTOF‐MS

Molecular characterization was performed by an ultra‐performance liquid chromatography quadrupole time‐of‐flight mass spectrometry (UPLC‐QTOF‐MS) technique (Q‐TOF 6540, Agilent). The mass range for all spectra was set to m/z 100–2000. All samples were dissolved in DMSO at a concentration of 10 mg mL^−1^.

### Characterization—NMR

NMR‐spectra were recorded on a Bruker AVAVCE III HD 700 MHz spectrometer. Chemicals shifts (δ) are quoted in ppm downfield of tetramethyl silane. The residual solvent signals were used as references for ^1^H, ^13^C, HSQC, H2BC, HMBC and HSQC‐TOCSY NMR spectra (DMSO‐d6: δH = 2.50 ppm, δC = 39.52 ppm). The samples were prepared at a concentration of 5 mg mL^−1^ for NMR experiments, excluding any additional descriptions.

2D diffusion‐ordered spectroscopy (DOSY) experiments were performed on a 400 MHz JEOL spectrometer using a 5 mm diameter indirect detection probe at 298 K. The bpp_dste_led_dosy sequence was used with nominal gradient strengths ranging from 20 to 280 mT m^−1^. A diffusion delay (Δ) and gradient lengths (δ) were set to 0.1 s and 2.0–9.0 ms, respectively, which depended on the samples. The dimension of their theoretical dimensions was calculated according to the Stokes–Einstein equation:

(1) $$D\;=\frac{k_{B}T}{6\pi \eta R}$$

where *D* is the diffusion coefficient, kB is the Boltzmann constant, *T* is the absolute temperature, *η* is the dynamic viscosity, and *R* is the radius of the spherical particle. The molecular weight was estimated based on the Stokes−Einstein Gierer–Wirtz Estimation according to ref. [48]

### Characterization—Optical Spectroscopy

UV–vis spectra were recorded at room temperature on Persee TU‐1901 UV–vis spectrophotometer. Fluorescence spectra were recorded on a F‐4600 (HITACHI) fluorescence spectrometer. All the spectra were recorded at room temperature using 10 mm path‐length cuvettes.

### Characterization—IR Spectroscopy

Fourier‐transform infrared spectroscopy was performed by the attenuated total reflection (ATR) method on a Nicolet iS50 (Thermos Scientific) equipped with a diamond plate and ZnSe lens.

### Characterization—Transmission Electron Microscopy (TEM)

TEM samples were prepared by dispersing as‐synthesized CPDs in DMSO or mixed solution, sonicating for 10 min, and then drop‐casting on to carbon coated copper grids. Samples were then imaged on a FEI Tecnai G2 F20 TEM at an acceleration voltage of 200 kV. The particles size distribution was calculated and measured by ImageJ software.

### Characterization—Fluorescence Quantum Yield and Lifetime (Time‐Correlated Single Photon Counting)

The absolute fluorescence quantum yields and lifetime were measured using a Edinburgh FLS 1000 Fluorescence Spectrometer fluorescence system with TCSPC mode and an integrated sphere, respectively.

### Characterization—Gel Permeation Chromatography

The GPC experiment was conducted using the FL‐GPC50 Integrated GPC/SEC System (Agilent). The distribution coefficient and selecting factor were set at 14.1000 and 0.7000, respectively. The linear function was employed to fit the standard curve.

### Characterization—Quantum Chemical Calculations

Geometry optimizations in the ground state were carried out within density functional theory (DFT) using the WB97XD functional combined with the 6–31G(d) basis set. Besides, time‐dependent density functional theory (TDDFT) has been used to compute the vertical absorption energy as well as to optimize the first excited electronic state, using the B3LYP functional and the 6–31G(d) basis set. All the calculations were performed with the Gaussian 16 suite of programs.^[^ ^30^ ^]^

All above the wave function analyses were conducted by using Multiwfn software,^[^ ^52^ ^]^ and the VMD 1.9.3 program.^[^ ^53^ ^]^ was used to plot the graph.

## Conflict of Interest

The authors declare no conflict of interest.

## Supporting information

Supporting Information

## Data Availability

The data that support the findings of this study are available in the supplementary material of this article.
