# Supplementary material for: The Synthesis of Functionalized Carbonized Polymer Dots via Reversible Assembly of Oligomers for Anti‐Counterfeiting, Catalysis, and Gas storage
Source: Adv Sci (Weinh). 2024 Aug 9;11(38):2405043. doi: 10.1002/advs.202405043 (PMC11481174; doi:10.1002/advs.202405043)
Supplement: Supplementary file 1 — Supporting Information [file ADVS-11-2405043-s001.docx]

Supplementary Information for:

**The Synthesis of Functionalized Carbonized Polymer Dots via Reversible Assembly of Oligomers for Anti-counterfeiting, Catalysis, and Gas storage**

Yu Wang, Yingxi Qin, Fengya Wang, Hongyu Zhang, Changxin Huangfu, Yushu Shi, Xize Chen, Zhenming Wang, Wenming Tian and Liang Feng*

^a^Department of Instrumentation and Analytical Chemistry, CAS Key Laboratory of Separation Science for Analytical Chemistry, Dalian Institute of Chemical Physics, Chinese Academy of Sciences, 457 Zhongshan Road, Dalian116023, P. R. China.

^b^State Key Laboratory of Molecular Reaction Dynamics and the Dynamic Research Center for Energy and Environmental Materials, Dalian Institute of Chemical Physics, Chinese Academy of Sciences, Dalian 116023, P. R. China

Keywords: *polymer carbon dots; selective gas storage; self-assembly; post-synthetic doping; customized functionalization*

Contents

[**1.** **Tables and Figures** 1](#_Toc171349150)

[1.1 Tables 1](#_Toc171349151)

[Table S1. 1](#_Toc171349152)

[Table S2. 3](#_Toc171349153)

[Table S3. 5](#_Toc171349154)

[Table S4. 6](#_Toc171349155)

[1.2 Figures 7](#_Toc171349156)

[Figure S1. 7](#_Toc171349157)

[Figure S2. 8](#_Toc171349158)

[Figure S3. 9](#_Toc171349159)

[Figure S4. 10](#_Toc171349160)

[Figure S5. 11](#_Toc171349161)

[Figure S6. 13](#_Toc171349162)

[Figure S7. 14](#_Toc171349163)

[Figure S8. 15](#_Toc171349164)

[Figure S9. 16](#_Toc171349165)

[Figure S10. 17](#_Toc171349166)

[Figure S11. 18](#_Toc171349167)

[Figure S12. 19](#_Toc171349168)

[Figure S13. 20](#_Toc171349169)

[Figure S14. 21](#_Toc171349170)

[Figure S15. 22](#_Toc171349171)

[Figure S16. 23](#_Toc171349172)

[Figure S17. 24](#_Toc171349173)

[Figure S18. 25](#_Toc171349174)

[Figure S19. 26](#_Toc171349175)

[Figure S20. 27](#_Toc171349176)

[Figure S21. 28](#_Toc171349177)

[Figure S22. 29](#_Toc171349178)

[Figure S23. 30](#_Toc171349179)

[Figure S24. 31](#_Toc171349180)

[Figure S25. 32](#_Toc171349181)

[Figure S26. 33](#_Toc171349182)

[Figure S27. 34](#_Toc171349183)

[Figure S28. 35](#_Toc171349184)

[Figure S29. 36](#_Toc171349185)

[Figure S30. 37](#_Toc171349186)

[Figure S31. 38](#_Toc171349187)

[Figure S32. 39](#_Toc171349188)

[Figure S33. 40](#_Toc171349189)

[Figure S34. 41](#_Toc171349190)

[Figure S35. 42](#_Toc171349191)

[Figure S36. 43](#_Toc171349192)

1. **Tables and Figures**

## Tables

### Table S1. List of the featured fragments of TM-H_2_O (positive mode).

| Experimental molecular weight  (m/z) | Molecular formula | Calculated molecular weight  (m/z) | Error  (ppm) | Relative abundance of ions  (%) | Suggested  structure |
| --- | --- | --- | --- | --- | --- |
| 109.0747 | C_6_H_8_N_2_ | 109.0760 | -11.9183 | 6.7047 | m-PD+H |
| 133.0742 | C_8_H_8_N_2_ | 133.0760 | -13.7140 | 2.9560 | *m*-PD+C_2_H_4_-4H |
| 147.0897 | C_9_H_10_N_2_ | 147.0917 | -13.4270 | 2.1650 | *m-*PD+(C_3_H_6_)-4H |
| 159.0896 | C_10_H_10_N_2_ | 159.0917 | -13.0428 | 10.2350 | *m-*PD+(C_4_H_8_)-4H |
| 170.0742 | C_10_H_7_N_3_ | 170.0713 | 17.2046 | 10.1837 | *m-*PD+(C_4_H_8_)+NH_3_-12H |
| 224.1154 | C_14_H_13_N_3_ | 224.1182 | -12.6005 | 8.1614 | 2(*m-*PD) -NH_3_+(C_2_H_4_) -4H |
| 231.0734 | C_12_H_10_N_2_O_3_ | 231.0764 | -13.0650 | 2.5273 | *m-*PD+TA-3H_2_O |
| 239.0783 | C_14_H_10_N_2_O_2_ | 239.0815 | -13.4013 | 1.4514 | (*m-*PD+TA-4H_2_O)+(C_2_H_4_)-2H |
| 249.0844 | C_12_H_12_N_2_O_4_ | 249.0870 | -10.37 | 100.0000 | *m-*PD+TA-2H_2_O |
| 267.0941 | C_12_H_14_N_2_O_5_ | 267.0989 | -12.9091 | 6.6686 | *m-*PD+TA-H_2_O |
| 277.1149 | C_14_H_16_N_2_O_4_ | 277.1183 | -12.2078 | 22.5352 | (*m-*PD+TA-2H_2_O)+C_2_H_4_ |
| 295.1251 | C_14_H_18_N_2_O_5_ | 295.1288 | -12.6995 | 6.7860 | (*m-*PD+TA-2H_2_O)+C_2_H_4_+H_2_O |
| 323.1562 | C_16_H_22_N_2_O_5_ | 323.1601 | -12.2169 | 3.5707 | (*m-*PD+TA-2H_2_O)+2(C_2_H_4_)+ H_2_O |
| 339.1409 | C_18_H_18_N_4_O_3_ | 339.1452 | -12.5816 | 3.9556 | 2(*m-*PD) +TA-3H_2_O |
| 389.0929 | C_18_H_16_N_2_O_8_ | 389.0979 | -12.9582 | 9.7082 | 2TA+m-PD-4H_2_O |
| 406.1193 | C_18_H_19_N_3_O_8_ | 406.1245 | -12.7818 | 7.9015 | 2TA+m-PD+NH_3_-4H_2_O |
| 407.1034 | C_18_H_18_N_2_O_9_ | 407.1085 | -12.5446 | 3.9040 | 2TA+m-PD-3H_2_O |
| 435.1344 | C_20_H_22_N_2_O_9_ | 435.1398 | -12.4359 | 3.6804 | (2TA+m-PD-3H_2_O)+C_2_H_4_ |
| 453.1446 | C_20_H_24_N_2_O_10_ | 453.1504 | -12.7353 | 1.1834 | (2TA+m-PD-3H_2_O)+C_2_H_4_+H_2_O |
| 479.1503 | C_24_H_22_N_4_O_7_ | 479.1561 | -12.1589 | 20.3985 | 2(TA+m-PD-2H_2_O)-H_2_O |
| 497.1605 | C_24_H_24_N_4_O_8_ | 497.1667 | -12.4506 | 12.0593 | (TA+m-PD-2H_2_O)+ (TA+m-PD-H_2_O)-H_2_O |
| 507.181 | C_26_H_26_N_4_O_7_ | 507.1874 | -12.6699 | 1.6311 | 2(TA+m-PD-2H_2_O)-H_2_O+C_2_H_4_ |
| 525.1915 | C_26_H_28_N_4_O_8_ | 525.1980 | -12.3572 | 3.5583 | 3(TA+m-PD-2H_2_O)-H_2_O+C_2_H_4_+H_2_O |
| 619.1594 | C_30_H_26_N_4_O_11_ | 619.1671 | -12.41 | 1.7847 | 3TA+2m-PD-7H_2_O |
| 637.1703 | C_30_H_28_N_4_O_12_ | 637.1776 | -11.5337 | 26.0351 | 3TA+2m-PD-6H_2_O |
| 655.1805 | C_30_H_30_N_4_O_13_ | 655.1882 | -11.7737 | 6.3144 | 3TA+2m-PD-5H_2_O |
| 665.2007 | C_32_H_32_N_4_O_12_ | 665.2089 | -12.4006 | 5.1697 | 3TA+2m-PD-6H_2_O+C_2_H_4_ |
| 683.2113 | C_32_H_34_N_4_O_13_ | 683.2195 | -12.0225 | 4.8946 | (3TA+2m-PD-6H_2_O+C_2_H_4_)+H_2_O |
| 709.2116 | C_36_H_32_N_6_O_10_ | 709.2253 | -19.2717 | 3.3279 | 3(TA+m-PD-2H_2_O)-2H_2_O |
| 727.2269 | C_36_H_34_N_6_O_11_ | 727.2358 | -12.2821 | 4.7432 | 2(TA+m-PD-2H_2_O)+(TA+m-PD-H_2_O)-2H_2_O |
| 867.2361 | C_42_H_38_N_6_O_15_ | 867.2468 | -12.3275 | 7.8121 | 4TA+3m-PD-9H_2_O |
| 885.2465 | C_42_H_40_N_6_O_16_ | 885.2574 | -12.2631 | 3.6626 | 4TA+3m-PD-8H_2_O |
| 895.2671 | C_44_H_42_N_6_O_15_ | 895.2781 | -12.2766 | 1.1362 | (4TA+3m-PD-9H_2_O)+C_2_H_4_ |
| 913.2771 | C_44_H_45_N_8_O_16_ | 913.2887 | -12.6532 | 1.9343 | (4TA+3m-PD-9H_2_O)+C_2_H_4_+H_2_O |
| 957.2928 | C_46_H_44_N_8_O_14_ | 957.3050 | -12.7180 | 1.0245 | 2(TA+m-PD-2H_2_O)+2(TA+m-PD-H_2_O)-3H_2_O |
| 1097.3019 | C_54_H_48_N_8_O_18_ | 1097.3159 | -12.7885 | 1.0490 | 5TA+4m-PD-12H_2_O |
|  |  |  |  |  |  |
|  |  |  |  |  |  |

### Table S2. List of the featured fragments of TM-EtOH (positive mode).

| Experimental molecular weight  (m/z) | Molecular formula | Calculated molecular weight  (m/z) | Error  (ppm) | Relative abundance of ions  (%) | Suggested  structure |
| --- | --- | --- | --- | --- | --- |
| 109.0752 | C_6_H_8_N_2_ | 109.0760 | -7.3343 | 7.3348 | m-PD+H |
| 133.0749 | C_8_H_8_N_2_ | 133.0760 | -8.2660 | 2.4608 | *m*-PD+C_2_H_4_-4H |
| 135.0905 | C_8_H_10_N_2_ | 135.0917 | 1.4804 | 2.4452 | *m*-PD+C_2_H_4_-2H |
| 147.0904 | C_9_H_10_N_2_ | 147.0917 | 8.8381 | 1.5817 | *m-*PD+(C_3_H_6_)-4H |
| 159.0906 | C_10_H_10_N_2_ | 159.0928 | -13.6524 | 49.8940 | *m-*PD+(C_4_H_8_)-4H |
| 170.0763 | C_10_H_7_N_3_ | 170.0713 | 29.3994 | 2.6301 | *m-*PD+(C_4_H_8_)+NH_3_-12H |
| 187.0585 | C_8_H_10_O_5_ | 187.0612 | -14.4177 | 10.9372 | TA-2H_2_O+C_2_H_4_ |
| 215.0899 | C_10_H_14_O_5_ | 215.0925 | -12.0739 | 41.7587 | TA-2H_2_O+2C_2_H_4_ |
| 224.1164 | C_14_H_13_N_3_ | 224.1182 | -8.0315 | 18.7459 | 2(*m-*PD) -NH_3_+(C_2_H_4_) -4H |
| 233.1001 | C_10_H_16_O_6_ | 233.1020 | -8.1509 | 18.2810 | TA-H_2_O+2C_2_H_4_ |
| 249.0844 | C_12_H_12_N_2_O_4_ | 249.0870 | -8.4307 | 3.4294 | *m-*PD+TA-2H_2_O |
| 261.1317 | C_12_H_20_O_6_ | 261.1343 | -10.1940 | 55.77362 | TA+3C_2_H_4_ |
| 277.1200 | C_14_H_16_N_2_O_4_ | 277.1183 | 6.1346 | 100.0000 | (*m-*PD+TA-2H_2_O)+C_2_H_4_ |
| 283.113 | C_14_H_18_O_6_ | 283.1176 | -20.1752 | 7.5340 | TA+4C_2_H_4_-6H |
| 295.1266 | C_14_H_18_N_2_O_5_ | 295.1288 | -7.4544 | 10.6190 | (*m-*PD+TA-H_2_O)+C_2_H_4_ |
| 303.1315 | C_16_H_18_N_2_O_4_ | 303.1350 | -11.6483 | 6.2327 | (*m-*PD+TA-2H_2_O)+2C_2_H_4_-2H |
| 305.1473 | C_16_H_20_N_2_O_4_ | 305.1507 | -11.0797 | 14.0571 | (*m-*PD+TA-2H_2_O)+2C_2_H_4_ |
| 323.1591 | C_16_H_22_N_2_O_5_ | 323.1601 | -3.0944 | 61.79171 | (*m-*PD+TA-2H_2_O)+2(C_2_H_4_)+ H_2_O |
| 327.1313 | C_18_H_18_N_2_O_4_ | 327.1350 | -11.4051 | 4.3283 | (*m-*PD+TA-2H_2_O)+3C_2_H_4_-6H |
| 339.1426 | C_18_H_18_N_4_O_3_ | 339.1452 | 7.6664 | 2.7399 | 2(*m-*PD) +TA-3H_2_O |
| 351.1887 | C_20_H_22_N_4_O_2_ | 351.1827 | 17.2275 | 2.5357 | 2(*m-*PD) +TA-4H_2_O+ C_2_H_4_ |
| 369.1991 | C_20_H_24_N_4_O_3_ | 369.1921 | 15.9429 | 2.9041 | 2(*m-*PD) +TA-3H_2_O+ C_2_H_4_ |
| 373.1728 | C_20_H_24_N_2_O_5_ | 373.1769 | -10.9733 | 3.1343 | (*m-*PD+TA-H_2_O)+4(C_2_H_4_)-6H |
| 385.1839 | C_20_H_24_N_4_O_4_ | 385.1881 | -10.9791 | 1.8770 | 2(*m-*PD) +TA-2H_2_O+C_2_H_4_ |
| 438.1989 | C_24_H_27_N_3_O_5_ | 438.2034 | -10.3719 | 4.2801 | [2(*m-*PD) -NH_3_+(C_2_H_4_) -4H]+TA-H_2_O+2C_2_H_4_ |
| 445.1571 | C_22_H_25_N_2_O_8_ | 445.1616 | -10.1963 | 5.8687 | [ (*m-*PD) +2TA-4H_2_O+2C_2_H_4_ |
| 462.1835 | C_22_H_27_N_3_O_8_ | 462.1882 | -10.1431 | 4.3098 | (*m-*PD+TA-2H_2_O)+TA-2H_2_O+NH_3_+2C_2_H_4_-2H |
| 491.1990 | C_24_H_30_N_2_O_9_ | 453.1504 | -9.1693 | 16.6267 | (*m-*PD+TA-2H_2_O)+TA-H_2_O +3C_2_H_4_ |
| 509.2101 | C_24_H_32_N_2_O_10_ | 509.2141 | -7.7944 | 35.1806 | (*m-*PD+TA-H_2_O)+TA-H_2_O +3C_2_H_4_ |
| 537.241 | C_26_H_36_N_2_O_10_ | 537.2454 | -8.1322 | 27.1141 | (*m-*PD+TA-H_2_O)+TA-H_2_O +4C_2_H_4_ |
| 553.2258 | C_28_H_32_N_4_O_8_ | 552.2226 | -8.2931 | 25.7811 | 2(TA+m-PD-2H_2_O)+2C_2_H_4_ |
| 579.2409 | C_30_H_34_N_4_O_8_ | 579.2449 | -6.9055 | 6.7869 | 2(TA+m-PD-2H_2_O)+3C_2_H_4_-2H |
| 599.2666 | C_30_H_38_N_4_O_9_ | 599.2712 | -9.4314 | 2.4703 | (TA+m-PD-2H_2_O)+ (TA+m-PD-H_2_O)+3C_2_H_4_ |
| 625.2824 | C_32_H_40_N_4_O_9_ | 625.2879 | -8.8007 | 1.7419 | (TA+m-PD-2H_2_O)+ (TA+m-PD-H_2_O)+4C_2_H_4_-2H |
| 649.2819 | C_34_H_40_N_4_O_9_ | 649.2868 | -9.2455 | 1.2251 | (TA+m-PD-2H_2_O)+ (TA+m-PD-H_2_O)+5C_2_H_4_-6H |
| 693.2355 | C_34_H_36_N_4_O_12_ | 693.2402 | -8.4329 | 1.660 | 3TA+2m-PD-6H_2_O+C_2_H_4_ |
| 721.2672 | C_36_H_40_N_4_O_12_ | 721.2726 | -7.5505 | 26.5221 | 2(*m-*PD+TA-2H_2_O)+TA-2H_2_O +3C_2_H_4_ |
| 739.2771 | C_36_H_42_N_4_O_13_ | 739.2832 | -8.2661 | 5.3458 | (*m-*PD+TA-H_2_O)+(*m-*PD+TA-2H_2_O)+TA-2H_2_O +3C_2_H_4_ |
| 767.3087 | C_38_H_46_N_4_O_13_ | 767.3145 | -7.5732 | 22.0315 | (*m-*PD+TA-2H_2_O)+(*m-*PD+TA-H_2_O)+TA-2H_2_O +4C_2_H_4_ |
| 785.3183 | C_38_H_48_N_4_O_14_ | 785.3251 | -8.6283 | 2.3351 | 2(*m-*PD+TA-H_2_O)+TA-2H_2_O +4C_2_H_4_ |
| 813.3495 | C_40_H_52_N_4_O_14_ | 813.3564 | -8.4539 | 5.8627 | 2(*m-*PD+TA-H_2_O)+TA-2H_2_O +5C_2_H_4_ |
| 997.3751 | C_50_H_56_N_6_O_16_ | 997.3837 | -8.5754 | 3.0830 | 2(*m-*PD+TA-2H_2_O)+(*m-*PD+TA-H_2_O)+TA-3H_2_O +3C_2_H_4_ |
| 1043.4164 | C_52_H_62_N_6_O_17_ | 1043.4255 | -8.7385 | 1.8291 | 3(*m-*PD+TA-H_2_O)+TA-3H_2_O +4C_2_H_4_ |

### Table S3. List of the featured fragments of TM (positive mode).

| Experimental molecular weight  (m/z) | Molecular formula | Calculated molecular weight  (m/z) | Error  (ppm) | Relative abundance of ions  (%) | Suggested  structure |
| --- | --- | --- | --- | --- | --- |
| 158.0505 | C_6_H_7_NO_4_ | 158.0448 | 36.17 | 33.8526 | TA+NH_3_-2H_2_O |
| 249.0862 | C_12_H_12_N_2_O_4_ | 249.0870 | -3.2117 | 48.3281 | *m-*PD+TA-2H_2_O |
| 327.1001 | C_14_H_18_N_2_O_5_S | 327.1009 | -2.4457 | 11.1553 | *m-*PD+TA-2H_2_O+DMSO |
| 339.1441 | C_18_H_18_N_4_O_3_ | 339.1452 | -3.2434 | 47.1600 | 2(*m-*PD) +TA-3H_2_O |
| 400.1442 | C_20_H_22_N_3_O_4_S | 400.1326 | 29.11 | 4.6838 | [2(*m-*PD) +TA-3H_2_O]-NH_3_+DMSO |
| 323.1562 | C_16_H_22_N_2_O_5_ | 323.1601 | -12.2169 | 3.5707 | (*m-*PD+TA-2H_2_O)+2(C_2_H_4_)+ H_2_O |
| 339.1409 | C_18_H_18_N_4_O_3_ | 339.1452 | -12.5816 | 3.9556 | 2(*m-*PD) +TA-3H_2_O |
| 389.0929 | C_18_H_16_N_2_O_8_ | 389.0979 | -12.9582 | 9.7082 | 2TA+m-PD-4H_2_O |
| 406.1193 | C_18_H_19_N_3_O_8_ | 406.1245 | -12.7818 | 7.9015 | 2TA+m-PD+NH_3_-4H_2_O |
| 407.1034 | C_18_H_18_N_2_O_9_ | 407.1085 | -12.5446 | 3.9040 | 2TA+m-PD-3H_2_O |
| 435.1344 | C_20_H_22_N_2_O_9_ | 435.1398 | -12.4359 | 3.6804 | (2TA+m-PD-3H_2_O)+C_2_H_4_ |
| 453.1446 | C_20_H_24_N_2_O_10_ | 453.1504 | -12.7353 | 1.1834 | (2TA+m-PD-3H_2_O)+C_2_H_4_+H_2_O |
| 479.1503 | C_24_H_22_N_4_O_7_ | 479.1561 | -12.1589 | 20.3985 | 2(TA+m-PD-2H_2_O)-H_2_O |
| 497.1605 | C_24_H_24_N_4_O_8_ | 497.1667 | -12.4506 | 12.0593 | (TA+m-PD-2H_2_O)+ (TA+m-PD-H_2_O)-H_2_O |
| 507.181 | C_26_H_26_N_4_O_7_ | 507.1874 | -12.6699 | 1.6311 | 2(TA+m-PD-2H_2_O)-H_2_O+C_2_H_4_ |
| 939.293 | C_48_H_42_N_8_O_13_ | 939.2955 | -2.6690 | 91.6615 | 4(*m-*PD+TA-2H_2_O)-3H_2_O |
| 975.3143 | C_48_H_46_N_8_O_15_ | 975.3166 | -2.3582 | 11.5157 | 2(*m-*PD+TA-2H_2_O)+2(*m-*PD+TA-H_2_O)-3H_2_O |
| 1029.3496 | C_54_H_48_N_10_O_12_ | 1029.3536 | -3.9743 | 11.8386 | 4(*m-*PD+TA-2H_2_O)+ *m-*PD-4H_2_O |
| 1096.3288 | C_54_H_50_N_9_O_17_ | 1096.3330 | -3.8446 | -3.8446 | 4(*m-*PD+TA-2H_2_O)+ (*m-*PD+TA-H_2_O)+NH_3_-5H_2_O |
| 1097.3019 | C_54_H_48_N_8_O_18_ | 1097.3159 | -12.7885 | 1.0490 | 5TA+4m-PD-12H_2_O |
| 1169.3611 | C_60_H_52_N_10_O_16_ | 1169.3646 | -3.0350 | 60.4126 | 5(*m-*PD+TA-2H_2_O)-4H_2_O |
| 1205.3836 | C_60_H_56_N_10_O_18_ | 1205.3858 | -1.8077 | 5.4990 | (3TA+2m-PD-6H_2_O+C_2_H_4_)+H_2_O |
| 1259.4169 | C_66_H_58_N_12_O_15_ | 1259.4228 | -4.7109 | 14.9602 | 5(*m-*PD+TA-2H_2_O)+ *m-*PD-5H_2_O |
| 1326.3975 | C_66_H_59_N_11_O_20_ | 1326.4022 | -3.5110 | 12.0439 | 5(*m-*PD+TA-2H_2_O)+(*m-*PD+TA-H_2_O)+NH_3_-6H_2_O |
| 1399.4301 | C_72_H_62_N_12_O_19_ | 1399.4338 | -2.6375 | 3.6626 | 6(*m-*PD+TA-2H_2_O)-5H_2_O |
| 1489.4844 | C_78_H_68_N_14_O_18_ | 1489.4920 | -5.0856 | 6.6680 | 6(*m-*PD+TA-2H_2_O)+ *m-*PD-6H_2_O |
| 1556.4658 | C_78_H_69_N_13_O_23_ | 1556.4713 | -3.5330 | 5.6986 | 6(*m-*PD+TA-2H_2_O)+(*m-*PD+TA-H_2_O)+NH_3_-7H_2_O |
| 1629.4974 | C_84_H_72_N_14_O_22_ | 1629.4974 | -3.3961 | 14.6950 | 7(*m-*PD+TA-2H_2_O)-6H_2_O |
| 1719.5486 | C_90_H_78_N_16_O_21_ | 1719.5611 | -7.2693 | 1.5998 | 7(*m-*PD+TA-2H_2_O)+ *m-*PD-7H_2_O |
| 1786.5242 | C_90_H_79_N_15_O_26_ | 1786.5404 | -9.0908 | 2.6129 | 7(*m-*PD+TA-2H_2_O)+(*m-*PD+TA-H_2_O)+NH_3_-8H_2_O |
| 1859.5678 | C_96_H_82_N_16_O_25_ | 1859.5721 | -2.2995 | 5.3808 | 8(*m-*PD+TA-2H_2_O)-7H_2_O |

### Table S4. The absorption, emission energies and correlating oscillator strength of different structures based on DFT calculations.

| **Structures** |  | **Absorption** | |  | | **Emission** | |
| --- | --- | --- | --- | --- | --- | --- | --- |
|  |  | **Wavelength (nm)** | **Oscillator strength** |  | **Wavelength (nm)** | | **Oscillator strength** |
| **a-π-i** |  | 265.52 | 0.04290 |  | 386.14 | | 0.07010 |
| **i-π-i** |  | 312.48 | 0.01100 |  | | 455.09 | 0.02160 |
| **a-π-a** |  | 292.89 | 0.03230 |  | | 709.13 | 0.00800 |
| **i-π-i'** |  | 255.72 | 0.01580 |  | | 355.53 | 0.01580 |
| **a-π-i+a-π-i** |  | 316.71 | 0.01140 |  | | 562.69 | 0.00020 |

## Figures


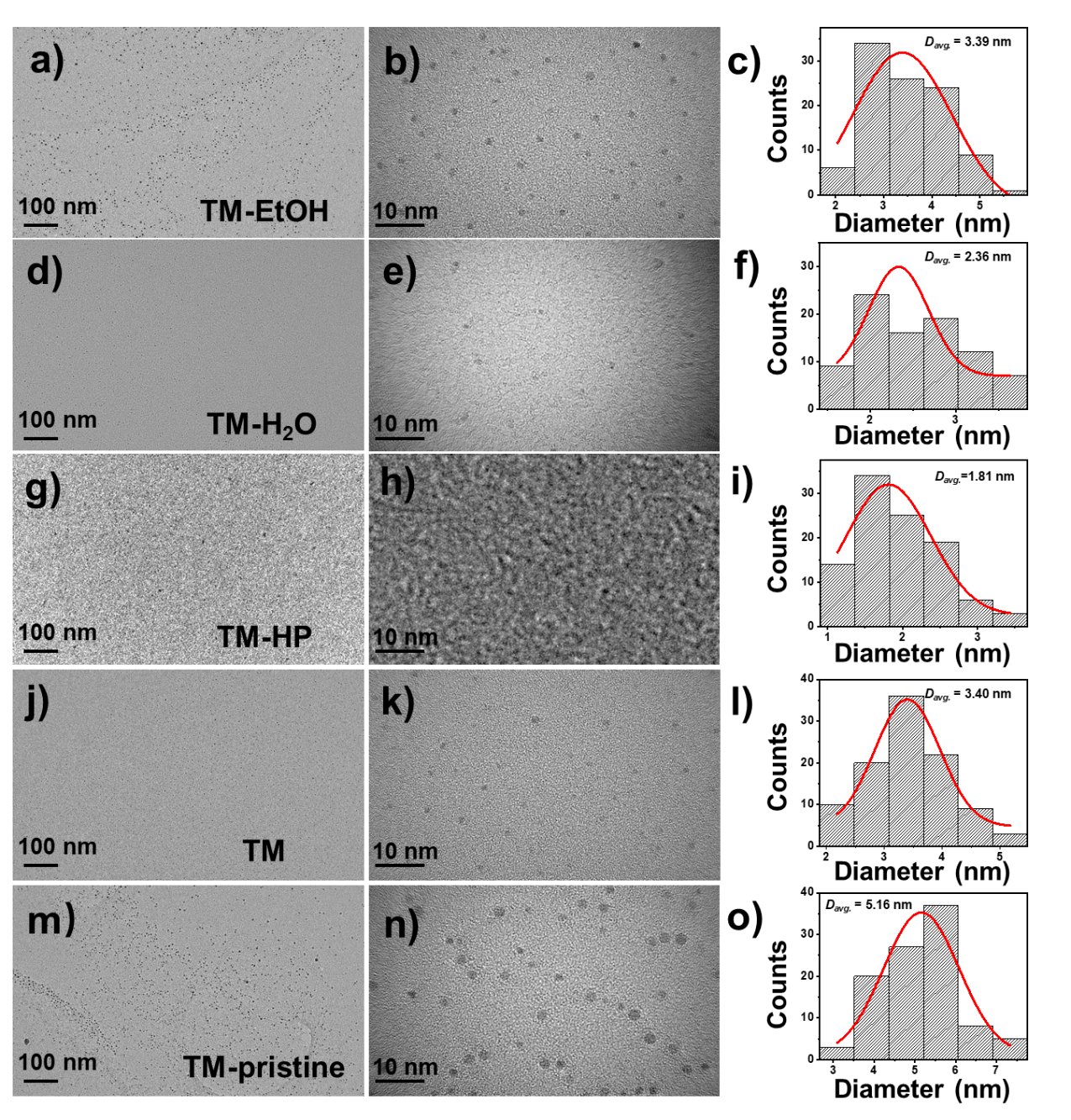


### Figure S1. TEM images and the corresponding particle distribution of a-c) TM-EtOH, d-f) TM-H_2_O, g-i) TM-HP, j-l) TM and m-o) TM-pristine.


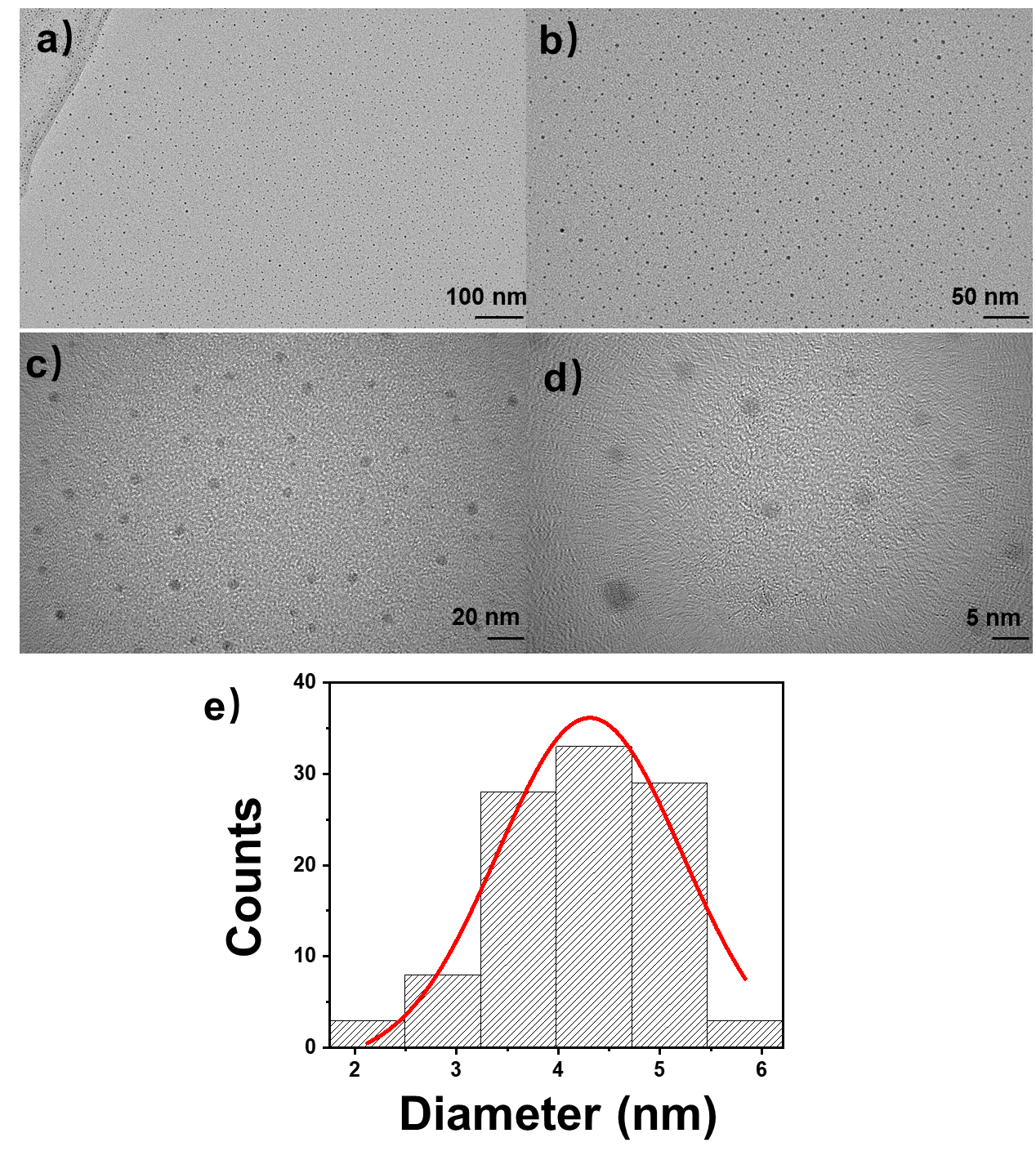


### Figure S2. TEM images of TM-mix and their particle distribution.


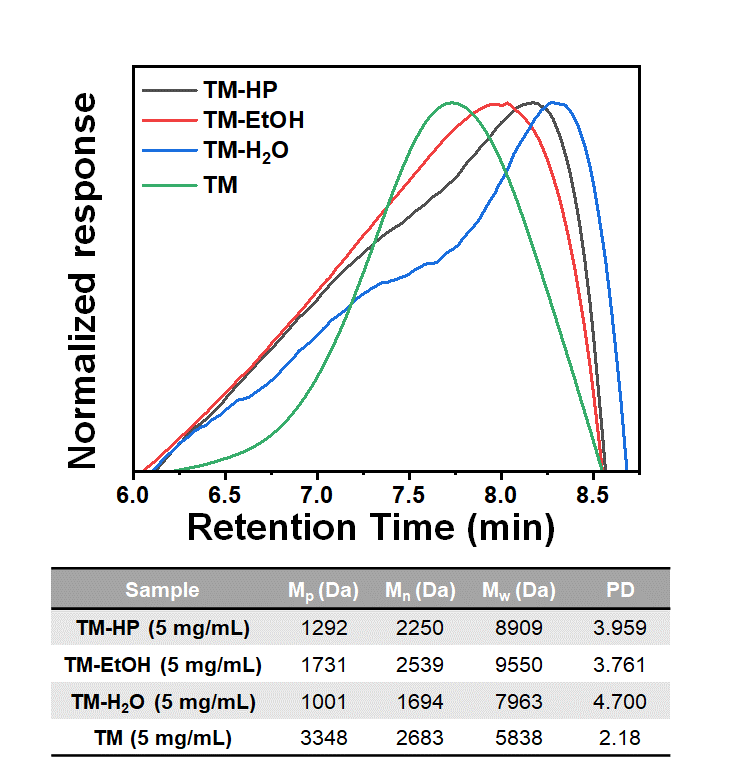


### Figure S3. The GPC results of four CPDs.

### Figure S4. HSQC NMR spectrum comparison of TM-EtOH, TM-H_2_O, TM-HP and TM. The color circle represents the C-H coupling associated with the structure of the same color.

### Figure S5. The DOSY NMR spectrum of four CPDs. The D value represented the diffusion coefficient of different species involved in each corresponding CPDs. Only TM exhibited a single measured D value. The following list presents the estimated dimension and molecular weight based on the Stokes-Einstein equation, as described in the method section.

### Figure S6. The HSQC NMR spectrum of TM-pristine, with the H and C signals annotated using distinct numerical and alphabetical labels that correspond to those present in structures of matching colors.

### Figure S7. The H2BC NMR spectrum of TM-pristine, with the H and C signals annotated using distinct numerical and alphabetical labels that correspond to those present in structures of matching colors.

### Figure S8. The high-solution H2BC NMR spectrum of TM-pristine, with the H and C signals annotated using distinct numerical and alphabetical labels that correspond to those present in structures of matching colors.

### Figure S9. The HMBC NMR spectrum of TM-pristine, with the H and C signals annotated using distinct numerical and alphabetical labels that correspond to those present in structures of matching colors.

### Figure S10. The high-resolution HMBC NMR spectrum of TM-pristine, with the H and C signals annotated using distinct numerical and alphabetical labels that correspond to those present in structures of matching colors.

### Figure S11. The HSQC NMR spectrum of TM, with the H and C signals annotated using distinct numerical and alphabetical labels that correspond to those present in structures of matching colors.

### Figure S12. The H2BC NMR spectrum of TM, with the H and C signals annotated using distinct numerical and alphabetical labels that correspond to those present in structures of matching colors. The inset spectrum illustrates the carbonyl C signal coupling with the adjacent methylene proton at (2.924, 168.765) ppm.

### Figure S13. The high-solution H2BC NMR spectrum of TM, with the H and C signals annotated using distinct numerical and alphabetical labels that correspond to those present in structures of matching colors.

### Figure S14. The HMBC NMR spectrum of TM, with the H and C signals annotated using distinct numerical and alphabetical labels that correspond to those present in structures of matching colors.

### Figure S15. The high-resolution HMBC NMR spectrum of TM, with the H and C signals annotated using distinct numerical and alphabetical labels that correspond to those present in structures of matching colors.


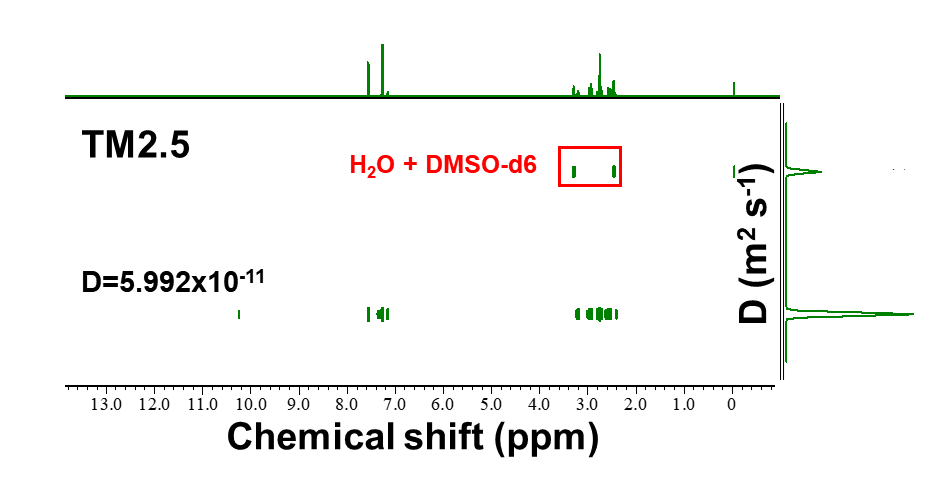


### Figure S16. DOSY NMR of TM2.5 in DMSO-*d*6.

### Figure S17. The HSQC NMR spectrum of TM0.4, with the H and C signals annotated using distinct numerical and alphabetical labels that correspond to those present in structures of matching colors.

### Figure S18. The H2BC NMR spectrum of TM0.4, with the H and C signals annotated using distinct numerical and alphabetical labels that correspond to those present in structures of matching colors.


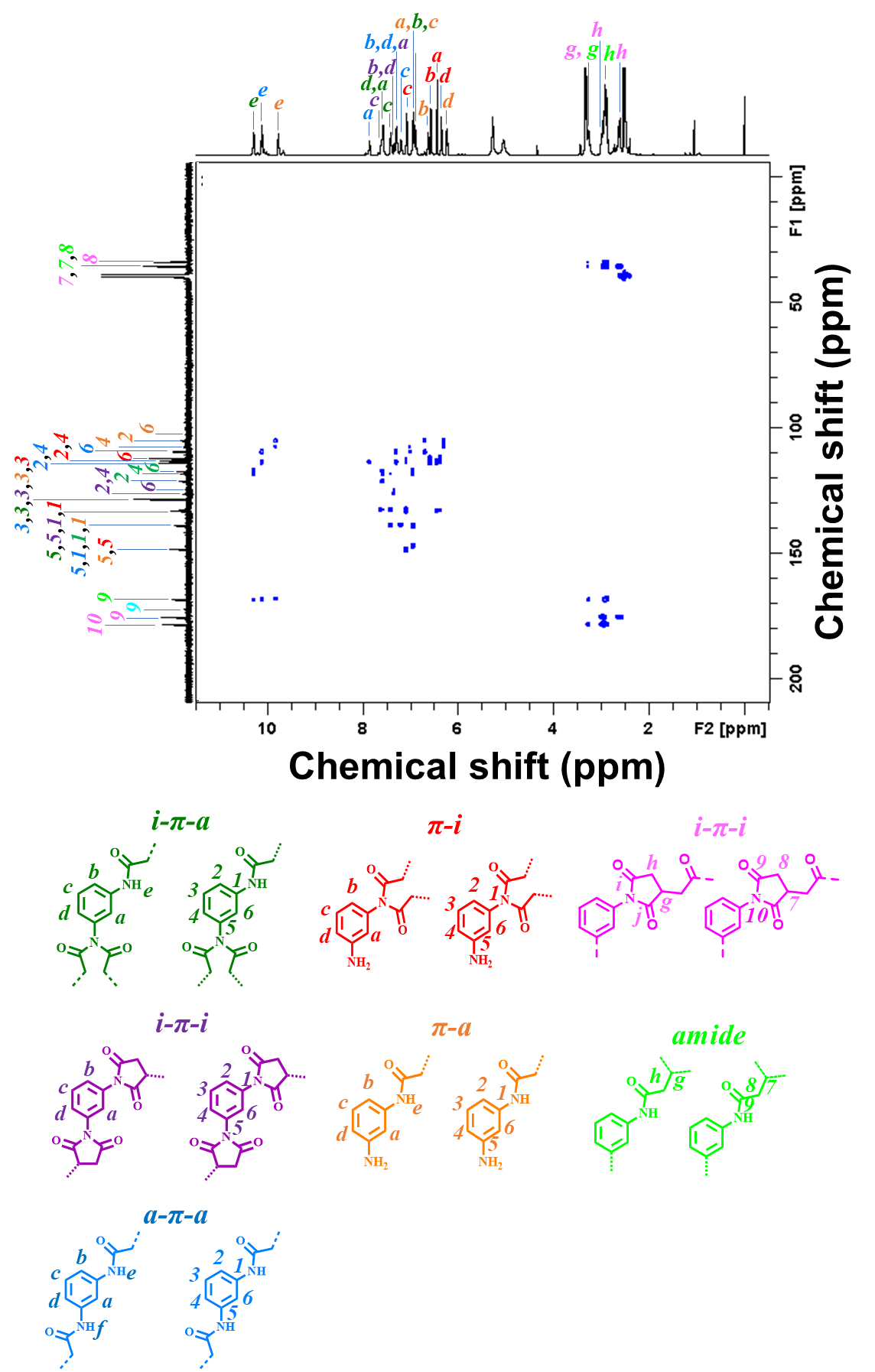


### Figure S19. The HMBC NMR spectrum of TM0.4, with the H and C signals annotated using distinct numerical and alphabetical labels that correspond to those present in structures of matching colors.


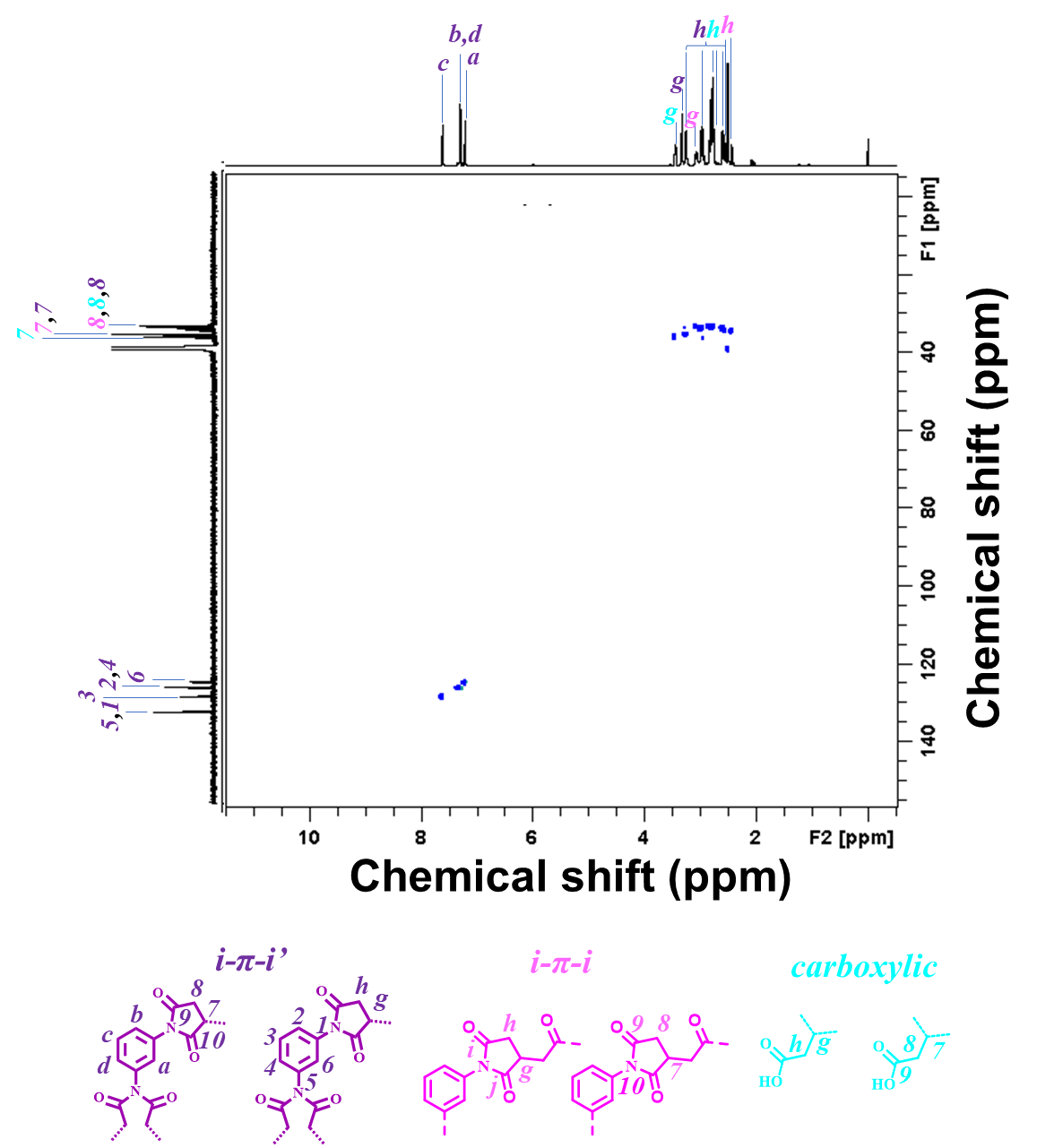


### Figure S20. The HSQC NMR spectrum of TM2.5, with the H and C signals annotated using distinct numerical and alphabetical labels that correspond to those present in structures of matching colors.


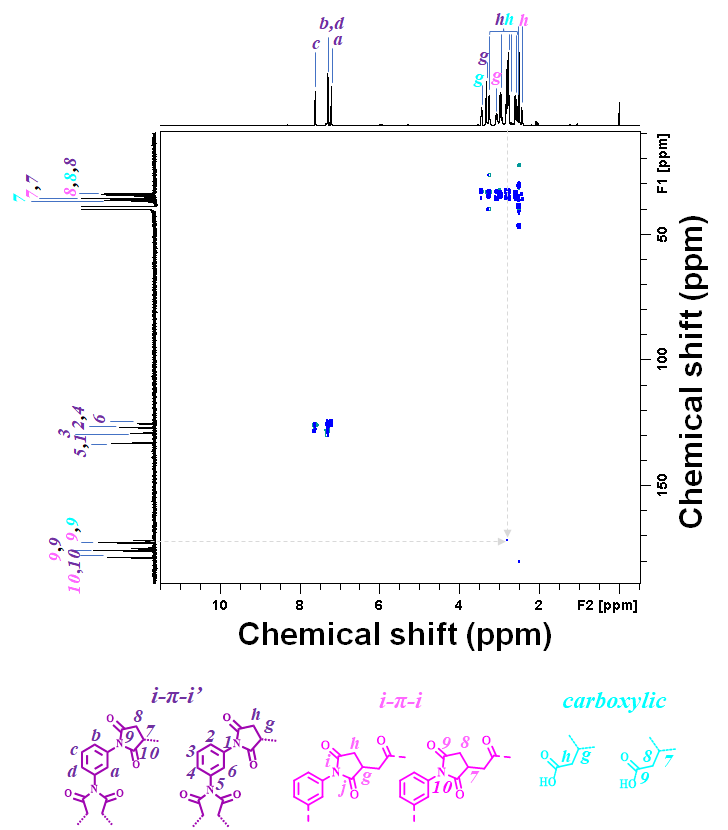


### Figure S21. The H2BC NMR spectrum of TM2.5, with the H and C signals annotated using distinct numerical and alphabetical labels that correspond to those present in structures of matching colors. The gray arrow showed carbonyl C signal coupling with methylene proton at (2.79, 172.45) ppm.


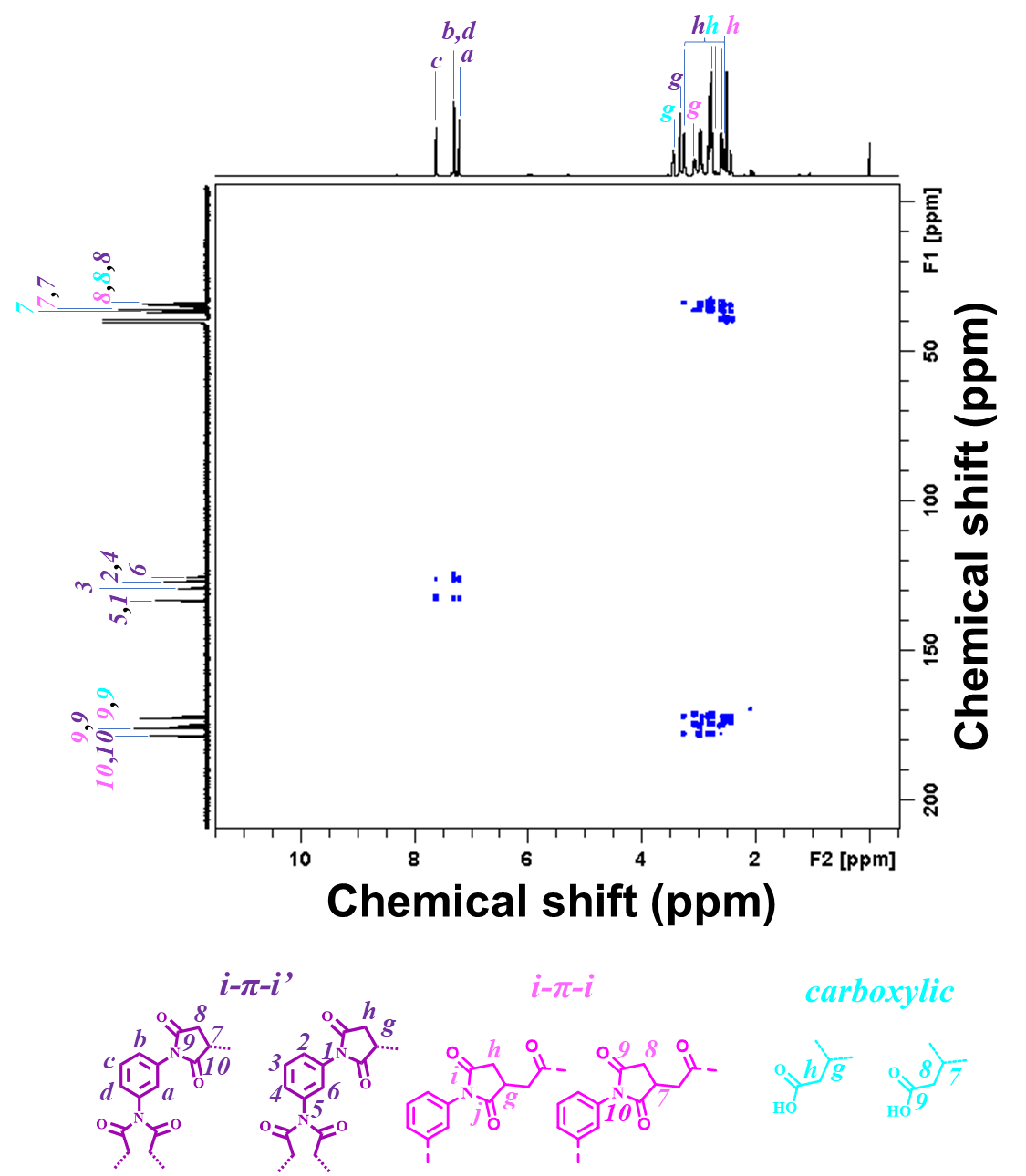


### Figure S22. The HMBC NMR spectrum of TM2.5, with the H and C signals annotated using distinct numerical and alphabetical labels that correspond to those present in structures of matching colors. The gray arrow showed carbonyl C signal coupling with methylene proton at (2.79, 172.45) ppm.


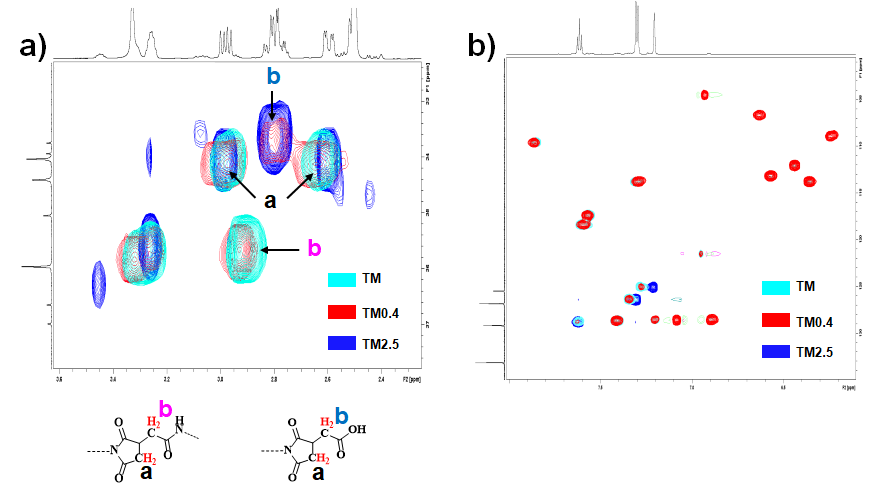


### Figure S23. The high-resolution HSQC NMR spectral comparison of TM0.4, TM and TM2.5, a) in methylene region, b) in phenyl C=C region, the i-π-i and i-π-i' are similarly observed in Figure 2e.

### Figure S24. The a) HMBC and b) HSQC-TOCSY NMR spectrum of TM2.5; c) the proposed three segments in polymer frameworks of TM2.5.

### Figure S25. The TEM images and the responding particle distribution of TM at the concentration of a-c) 1 mg/mL, d-f) 5 mg/mL, g-i) 37.5 mg/mL and j-l) 150 mg/mL.


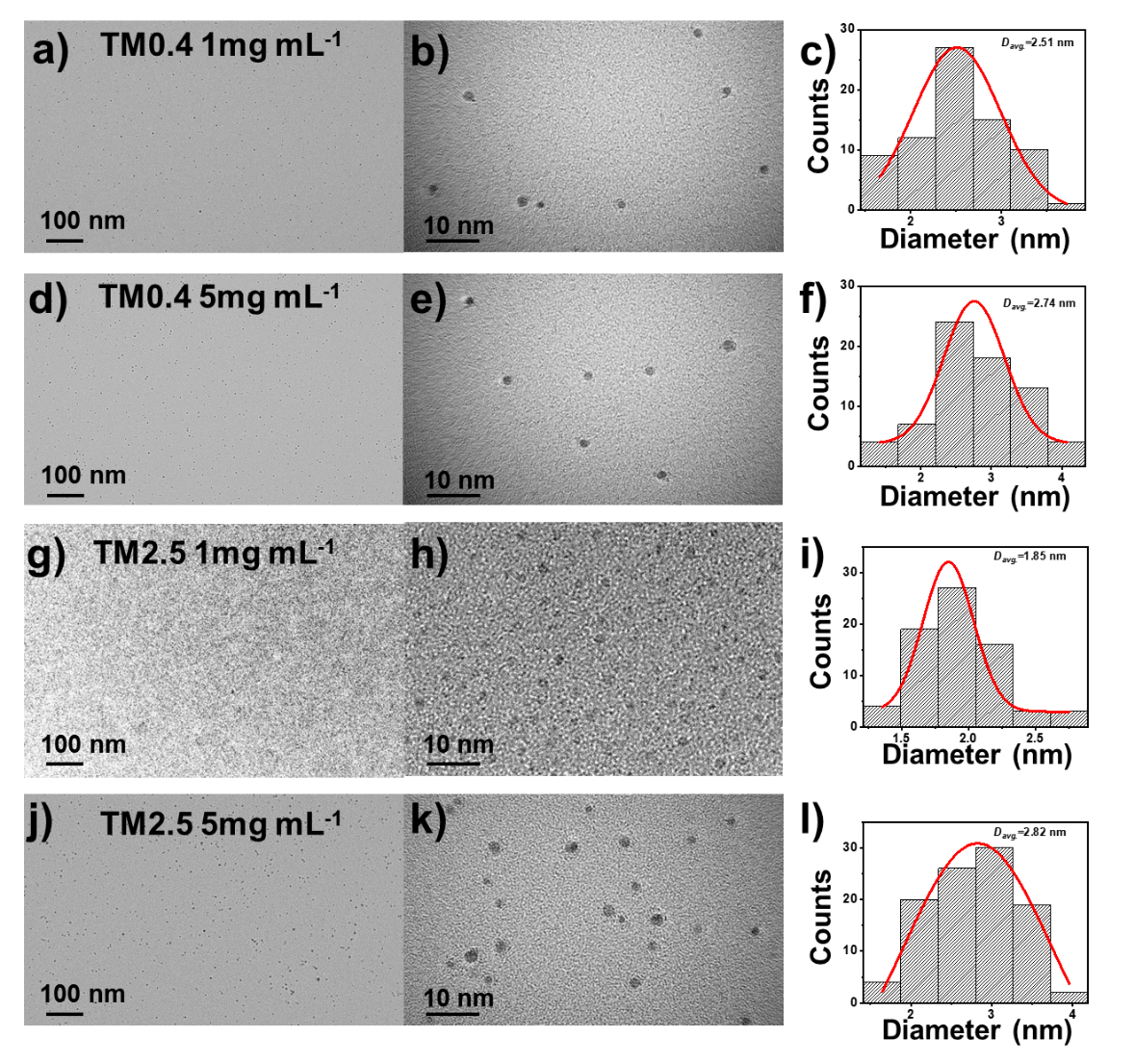


### Figure S26. TEM images and the corresponding particle distribution of a-c) TM0.4 1 mg mL^-1^, d-f) TM0.4 5 mg mL^-1^, g-i) TM2.5 1 mg mL^-1^, and j-l) TM2.5 5 mg mL^-1^


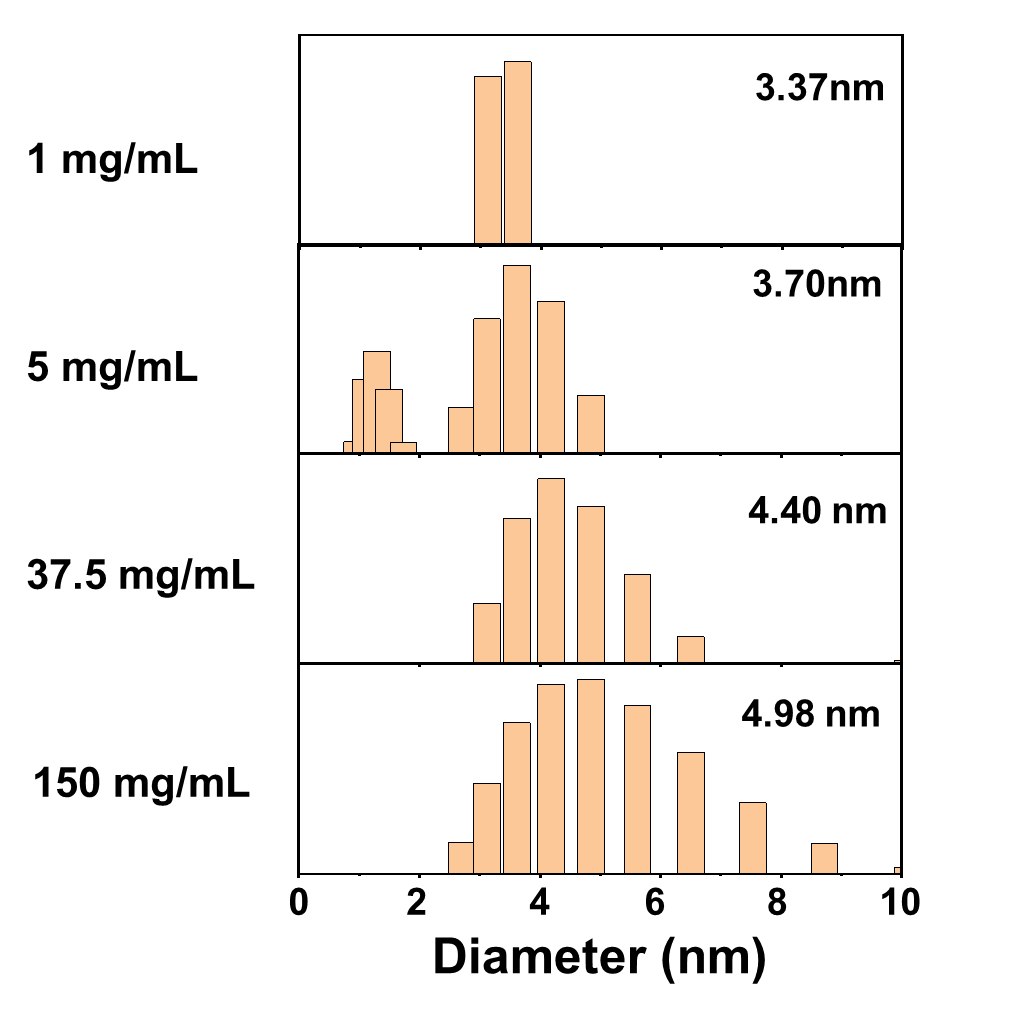


### Figure S27. DLS analysis of TM at different concentrations in DMSO solution.

### Figure S28. The MS results of TM at the concentration of a) 1 mg/mL and b) 5 mg/mL. c) The GPC result of TM at the concentration of 1 mg/mL and 5 mg/mL.

**
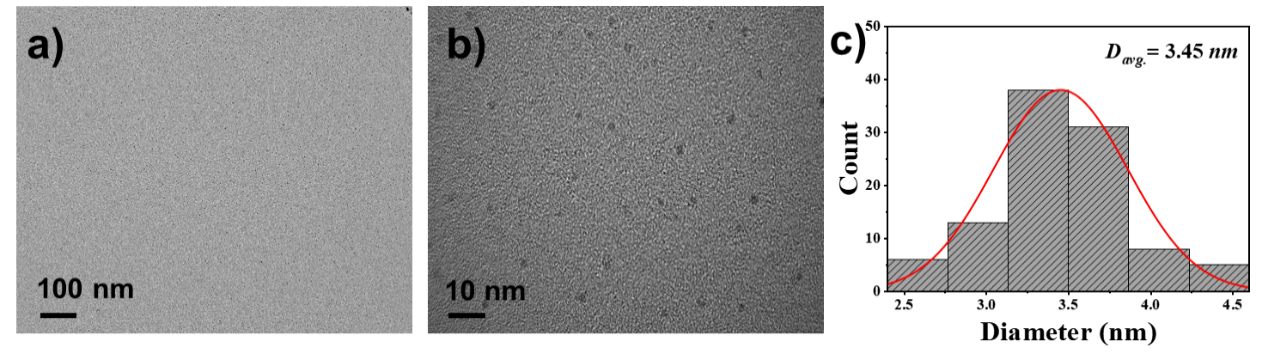
**

## Figure S29. TEM images and the corresponding particle distribution of TM at the concentration of 5 mg mL^-1^ which was prepared by diluting from a parent solution at the concentration of 150 mg mL^-1^


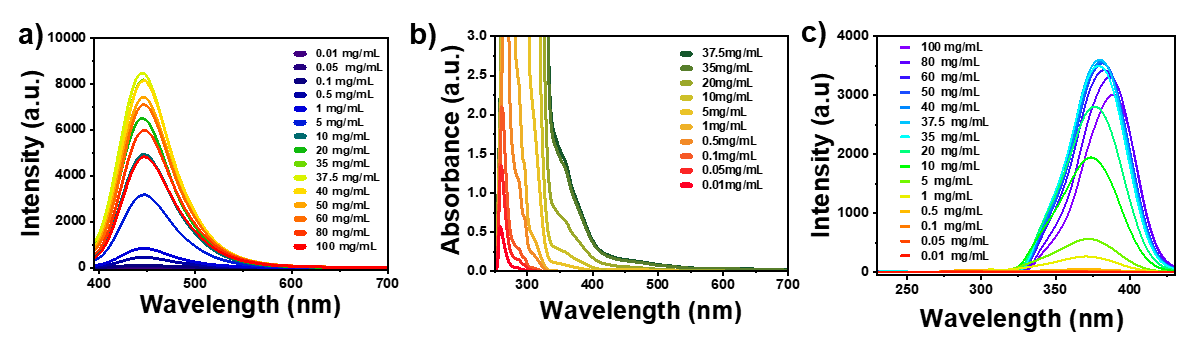


### Figure S30. The a) emission and b) absorption and c) excitation spectrum of TM in DMSO solution at varying concentrations.

###
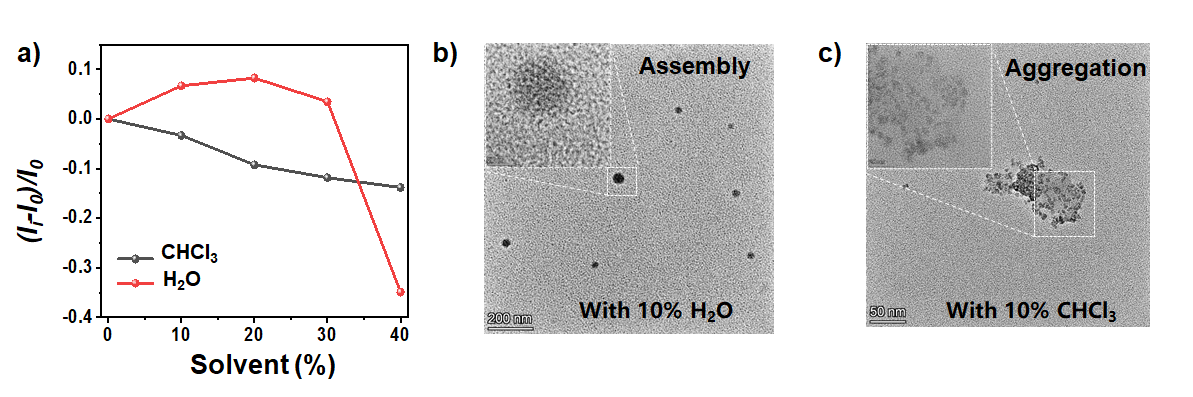


## Figure S31. a) The variation in TM emission intensity at 441 nm with a concentration of 5 mg/mL upon the addition of different percentages of H_2_O (red curve) and CHCl_3_ (black curve) in DMSO solutions. TEM images of TM (5 mg/mL) in the presence of b) 10% H_2_O and c) 10% CHCl_3_ in DMSO solution, illustrating the facilitation of self-assembling to larger particles in the presence of 10% H_2_O, while the generation of particle aggregation in the presence of 10% CHCl_3_.


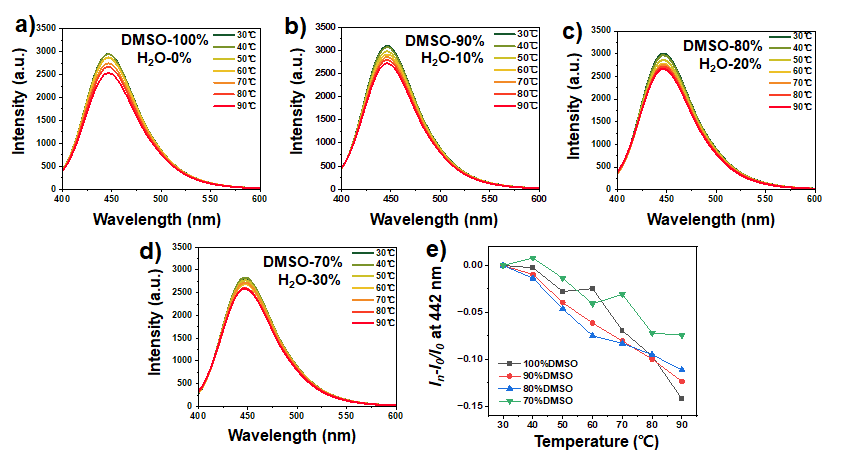


### Figure S32. The variation in TM emission intensity of different temperature at 442 nm with a concentration of 5 mg/mL upon the addition of a) 0% H_2_O, b) 10% H_2_O, c) 20% H_2_O, d) 30% H_2_O. e) The change in emission intensity curve at λ_441_ was investigated upon heating to 90 ℃ in the presence of varying proportions of H_2_O in DMSO solutions of TM.


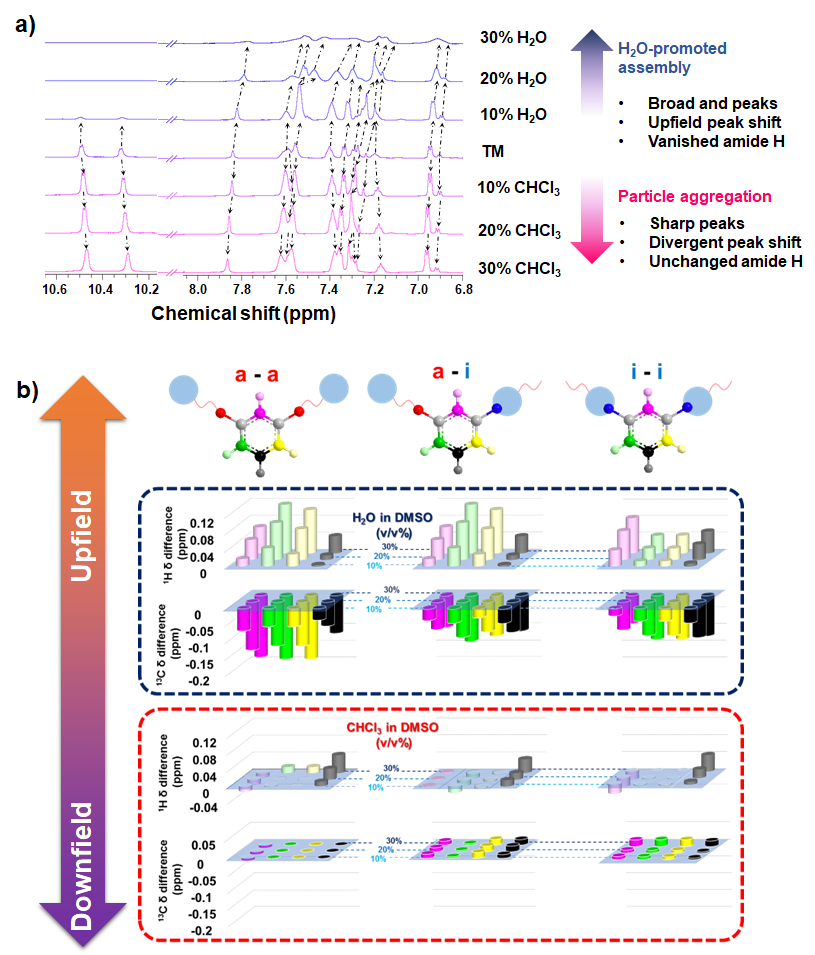


### Figure S33. a) The corresponding 1H NMR of spectral comparison, highlighting the different changes of protons chemical environment in TM during the assembly and the aggregation process. b) summarized the change (δ) of chemical shift at phenyl ring of TM in DMSO-d_6_ solution in the presence of H_2_O and CHCl_3_ at varying percentages, according to HSQC NMR. The proton and carbon signals exhibit significant changes in the presence of H_2_O, whereas their changes are minimal in the presence of CHCl_3_


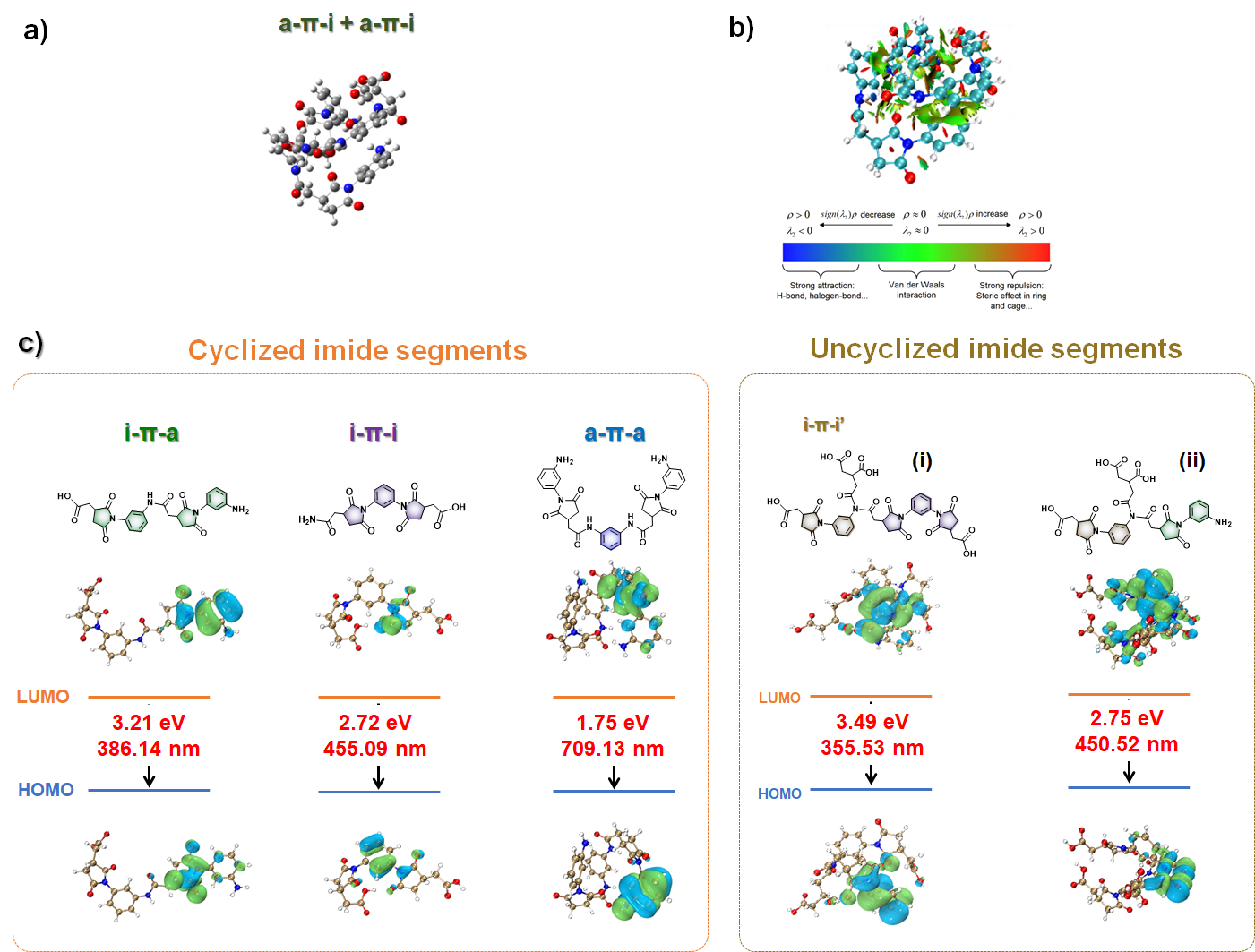


### Figure S34. a) The optimized structure of two a-π-i segments (a-π-i + a-π-i). b) The reduced density gradient (RGD) structure of two a-π-i segments (a-π-i + a-π-i). c) The LUMO, HOMO molecular orbitals and emission energies of different segments involved in TM polymer frameworks, e) the cyclized imide segments and f) the cyclized imide segments, calculated based on DFT using the WB97XD functional combined with the 6-31G(d) basis set.


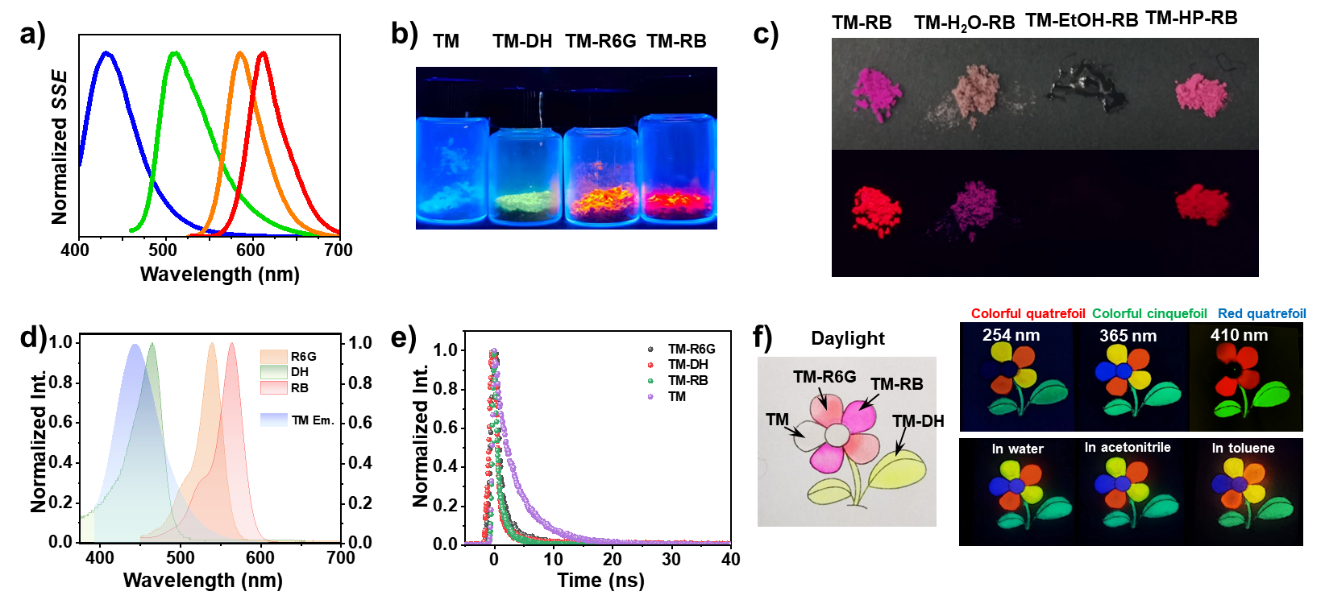


### Figure S35. a) The emission spectrum of different fluorophore-doped TM and b) corresponding photograph. c) photograph of TM-RB comparing to other RB-doped CPDs synthesized by conventional one-pot synthetic methods, under daylight(top) and UV light(bottom). d) spectral overlapping between TM emission and the absorption of different fluorescent dyes. e) TCSQC fluorescence decay comparison of TM and different fluorophore-doped TM. f) Photographs of TM doped with different fluorophores: rhodamine B (RB), rhodamine 6G (R6G), and 3,6-diaminoacridine hydrochloride (DH).


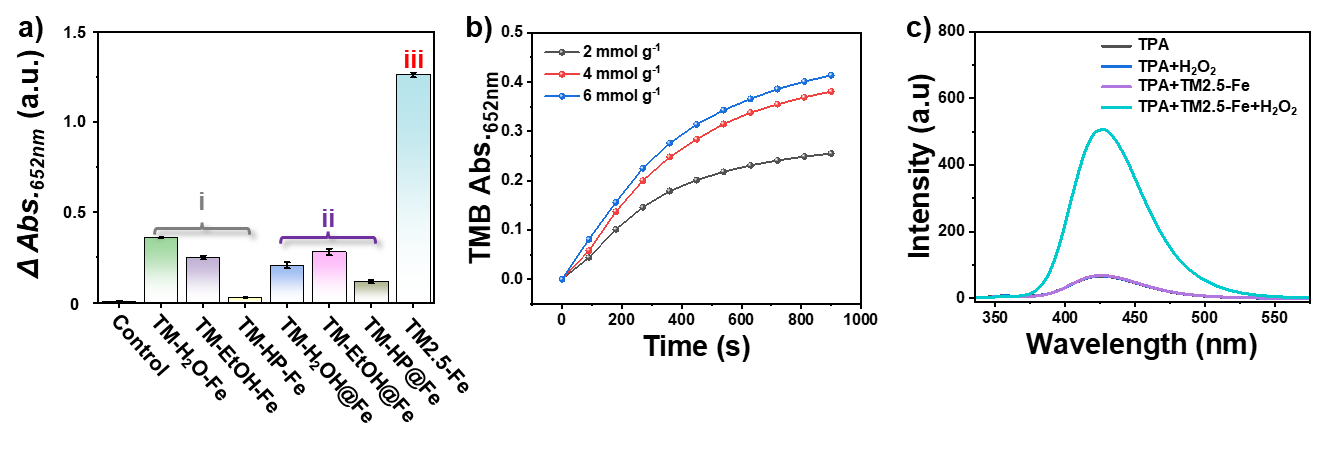


## Figure S36. a) Catalytic oxidation performance of CPDs synthesized using different methods illustrated in Scheme 1. b) The absorbance changes of a 0.1 mM TMB aqueous solution in the presence of TM2.5-Fe doped at varying Fe^3+^ concentrations. c) The fluorescence intensity of TPA in the absence/presence of H_2_O_2_ and TM2.5-Fe (6mmol g^-1^), respectively.
